# AP4 modulated by the PI3K/AKT pathway promotes prostate cancer proliferation and metastasis of prostate cancer via upregulating L-plastin

**DOI:** 10.1038/cddis.2017.437

**Published:** 2017-10-05

**Authors:** Changhao Chen, Qingqing Cai, Wang He, Thomas B Lam, Jianxun Lin, Yue Zhao, Xu Chen, Peng Gu, Hao Huang, Miaoxin Xue, Hao Liu, Feng Su, Jian Huang, Jianping Zheng, Tianxin Lin

**Affiliations:** 1 Department of Urology, Sun Yat-sen Memorial Hospital, Guangzhou, China; 2 Guangdong Provincial Key Laboratory of Malignant Tumor Epigenetics and Gene Regulation, Sun Yat-Sen Memorial Hospital, Sun Yat-Sen University, Guangdong, China; 3 Department of Internal Medicine, Sun Yat-Sen University Cancer Center, Guangzhou, China; 4 Academic Urology Unit, University of Aberdeen, Cornhill Road, Aberdeen, UK; 5 Department of Tumor Intervention, First Affiliated Hospital of Sun Yat-Sen University, Guangzhou, China; 6 Mosaic Laboratories, Lake Forest, CA, USA

## Abstract

The transition from androgen-dependent to metastatic castration-resistant prostate cancer (PCa) is a lethal event of uncertain molecular aetiology. Our previous studies demonstrated that L-plastin is involved in PCa invasion and metastasis and is upregulated by androgen and oestrogen in the hormone-dependent PCa cell line LNCaP. We recently found that L-plastin expression is consistently activated even after androgen deprivation, suggesting that androgen-independent transcription factors may regulate its expression. Herein, we performed sequential deletion and luciferase analysis of the L-plastin promoter and found that an androgen-independent regulatory factor prominently located in the region close to the transcription initiation site (−216 to +118) may facilitate L-plastin upregulation. AP4 was then identified as the relevant transcription activator that directly binds to the L-plastin promoter, as confirmed by EMSAs, supershift assays and CHIP-qPCR experiments. Moreover, we determined that the AP4/L-plastin axis is regulated by the phosphatidylinositol 3-kinase (PI3K)/AKT pathway, contributing to PCa metastasis and castration resistance. Furthermore, we found that AP4 promotes PCa metastasis by upregulating L-plastin expression *in vitro* and *in vivo*. We collected a total of 136 PCa tissues and corresponding adjacent normal tissues from patients who underwent prostatectomy at Sun Yat-Sen Memorial Hospital from 2005 to 2015 and measured AP4 and L-plastin protein levels by immunohistochemistry. The results showed that AP4 levels strongly correlated with those of its downstream target gene L-plastin, were significantly upregulated in PCa tissues, were positively correlated with lymph node metastasis and Gleason scores over 7, and were an independent prognostic factor for patient survival. In summary, these findings support a plausible mechanism by which the AP4/L-plastin axis is regulated by the PI3K/AKT pathway in human PCa and may represent a novel therapeutic target in PCa treatment.

Prostate cancer (PCa), the second most common malignant tumour in men worldwide,^1^ and proceeds through a series of defined states classified as prostatic intraepithelial neoplasia, cancer *in situ*, and metastatic cancer.^2^ Most metastatic cases of PCa ultimately develop castration resistance,^3^ the management of which remains a considerable challenge. The phosphatidylinositol 3-kinase (PI3K)/AKT pathway is an intracellular signalling cascade that has an important function in apoptosis, malignant transformation, and tumour progression and metastasis.^4, 5, 6^ Indeed, PI3K/AKT pathway is frequently activated in PCa, and it has been demonstrated to play crucial roles in the progression of castration-resistant prostate cancer (CRPC).^7, 8^ Alterations in PI3K/AKT pathway components occur in 42% of primary prostate tumours and 100% of metastatic prostate tumours.^9^ Owing to their important roles during progression to CRPC, components of the PI3K/AKT pathway are currently considered promising targets in the treatment of CRPC patients.^10, 11^

Although the isoform of L-plastin expressed in haematopoietic cell lineages is not active in most normal cells, it is ectopically activated and upregulated in various types of solid malignant tumours in humans.^12, 13^ Overexpression of L-plastin is involved in PCa invasion and metastasis both *in vitro* and *in vivo*.^14, 15^ We previously demonstrated that L-plastin is upregulated by both oestrogen and androgen exposure in a hormone-sensitive PCa model and is associated with a malignant state in prostatic epithelial cells.^16^ Moreover, we recently noted that androgen-insensitive PCa cells (LNCaP-AI and PC-3 cells) overexpress L-plastin, suggesting that other non-steroid-dependent factors may promote expression of the protein. However, little is known about the mechanisms responsible for regulating L-plastin expression or the elements that participate in this regulation in CRPC.

AP4/TFAP4/AP-4 is a ubiquitously expressed basic helix-loop-helix leucine-zipper (bHLH-LZ) transcription factor that forms homodimers that bind to the consensus E-box motif 5-CAGCTG-3.^17^ Unlike other HLH proteins, AP4 contains two additional dimerization motifs consisting of the leucine repeat elements LR1 and LR2.^18^ Previous studies reported that AP4 expression was positively correlated with survival and distant metastasis in two different colorectal cancer patient cohorts and that AP4 overexpression was associated with poor patient prognosis in gastric^19^ and liver cancer.^20^ However, the biological roles and clinical significance of AP4 and its downstream target genes in CRPC remain unclear.

In the present study, we aimed to identify the association of AP4 with castration resistance in PCa and analysed its correlation with PCa patient clinicopathological characteristics and prognosis. We found that AP4 is a key transcription factor that directly binds to the L-plastin promoter and increases L-plastin expression in PCa cells. We also showed that the AP4/L-plastin axis is regulated by the PI3K/AKT pathway, which contributes to PCa metastasis and castration resistance. Taken together, our findings strongly suggest that AP4 is involved in PCa, is a potential diagnostic and prognostic marker for the disease and may be a promising therapeutic target in improved PCa treatment.

## Results

### Establishment of an androgen-independent LNCaP subline

**Figure 1:**
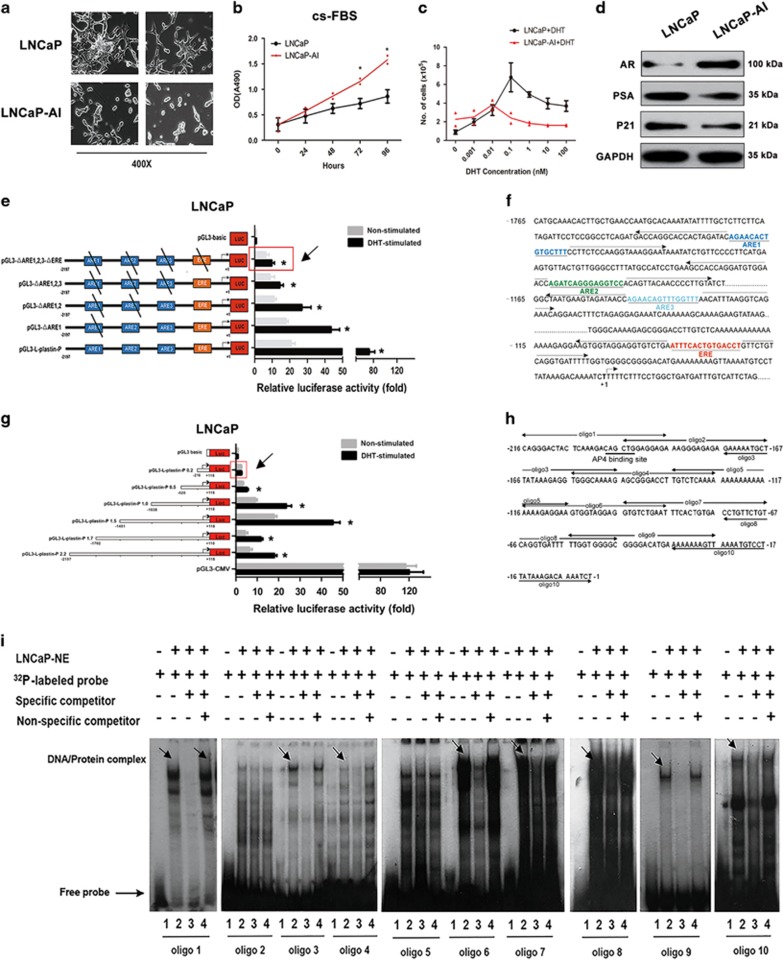
Identification of the androgen-independent elements responsible for upregulation of L-plastin. (**a**) the × 400 photographs of LNCaP and LNCaP-AI cells. (**b**) MTT assays showed the proliferation of LNCaP and LNCaP-AI cells in steroid-depleted medium (cs-FBS). (**c**) The dose–response curve of LNCaP-AI and LNCaP cells proliferation over a range of DHT concentration. (**d**) The expressions of AR, PSA and p21 were examined by western blot analyses in LNCaP and LNCaP-AI cells. (**e**,**f**) Transcriptional activity of the L-plastin promoter was evaluated by the sequentially deletions of the androgen and oestrogen elements into the LNCaP cell line by examining the L-plastin promoter linked to Renilla luciferase activity. (**g**) Deletion analysis of pGL3-ΔARE1,2,3-ΔERE which is the construct with deletions of both AREs and EREs. Schematic presentation of pGL3-L-plastin-P 0.2 which encompassed nucleotides −216 to +118 in L-plastin promoter. The pGL3-basic and pGL-3-CMV were transfected as negative control and positive control. (**h**,**i**) EMSA analyses were applied to identify androgen-independent response elements in the −216 bp fragment at 3′end of L-plastin promoter. Ten pairs of distinctive, overlapping oligoes in this area for synthesized and labelled with ^32^P. The arrows indicated positive band shift sites

We have previously demonstrated that L-plastin is a potential biomarker for PCa progression.^15, 21^ To determine whether L-plastin functions in CRPC progression, we established an androgen-independent LNCaP subline known as LNCaP-AI using previously described methods.^22, 23, 24^ LNCaP cells have an epithelial morphology and feature tapered unbranched processes; LNCaP-AI cells exhibit a neuronal morphology with compactly rounded cell bodies (Figure 1a). Proliferation of the two cell lines was compared by the MTT assay. The growth rate of LNCaP-AI cells in steroid-depleted medium was higher than that of LNCaP cells (Figure 1b). In addition, we performed a dose–response curve for LNCaP and LNCaP-AI cell proliferation over a range of dihydrotestosterone (DHT) concentrations. Both LNCaP-AI and LNCaP cells exhibited a biphasic response after incubation with DHT, with LNCaP-AI cells showing less proliferative stimulation by DHT treatment compared with LNCaP cells (Figure 1c). The qRT-PCR and western blot analyses indicated higher levels of AR expression in LNCaP-AI cells compared to LNCaP cells, though PSA levels were higher in LNCaP cells than in LNCaP-AI cells (Figure 1d and Supplementary Figure S1).

### Identification of the androgen-independent elements responsible for L-plastin upregulation

To elucidate the mechanisms by which L-plastin expression is regulated, we amplified a series of L-plastin-luc promoter constructs containing the indicated deletions (−2197/+118) from purified DNA of LNCaP and cloned these constructs into pGL3-basic vectors. Both androgen response elements (AREs) and oestrogen response elements (EREs) were deleted to produce these constructs. As indicated in Figure 1e, luciferase activity with the native L-plastin promoter (pGL3-L-plastin-P) was significantly increased by DHT stimulation. The pGL3-basic and pGL3-CMV plasmids served as negative and positive controls, respectively. Compared with the pGL3-basic plasmid, constructs containing ARE1, -2, -3 and ERE deletions (pGL3-ΔARE1,2,3-ΔERE) displayed significantly increased luciferase activity after DHT stimulation (Figures 1e and f). These findings suggest that L-plastin expression is regulated by androgen through transcription factors other than those that bind to the response elements indicated above.

To identify the key sequences responsible for L-plastin promoter activity, we performed exonuclease deletions at the 5′ end of pGL3-ΔARE1,2,3-ΔERE to produce sequential deletion constructs. Interestingly, the shortest construct (pGL3-L-plastin-P 0.2), which encompassed nucleotides −216 to +118, displayed relatively strong baseline luciferase activity but only limited responsiveness to DHT stimulation (Figure 1g). These results indicate that some androgen-independent transcription factor response elements located mainly near the transcription initiation site of the promoter may facilitate upregulation of L-plastin expression in the absence of DHT. We subsequently synthesized 10 pairs of distinctive, overlapping oligonucleotides specific for the 216-bp fragment at the 3′end of the L-plastin promoter and labelled them with ^32^P (Figure 1h). An electrophoretic mobility shift assay (EMSA) was then performed to identify the androgen-independent response elements in this region. Positive band shifts were observed for oligonucleotides 1, 6, 8 and 10 (Figure 1i). Taken together, these results indicate that androgen-independent transcription factors may function as activators of L-plastin expression.

### AP4 binds to its binding site within the L-plastin promoter in CRPC cells

**Figure 2:**
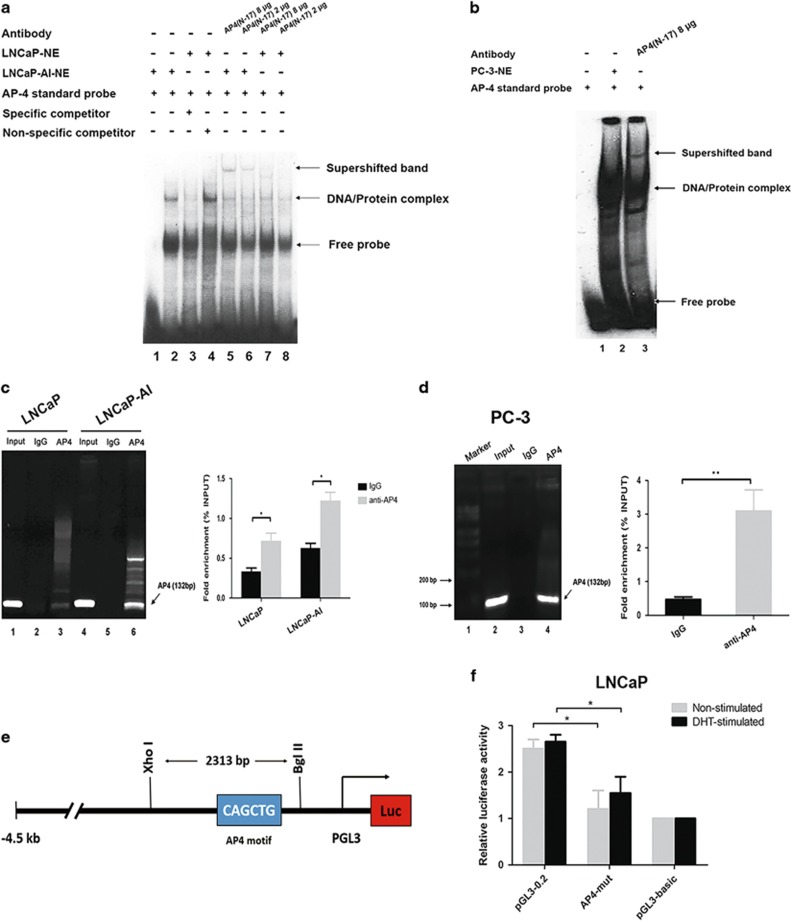
Identification of AP4 binding site in L-plastin promoter in PCa cells. (**a**,**b**) EMSA and supershift assays consensus AP4 probe containing the consensus AP4 binding site (CAGCTG) was used to validate the presence of the AP4 factor using the NE from LNCaP cells, LNCaP-AI cells and PC-3 cells. Both supershifted complexes and DNA/protein complexes were shown as an arrow head to the right of the panel. (**c**,**d**) The chromatin immunoprecipitation (ChIP) assay revealed that AP4 binds the L-plastin promoter. IgG was used as a negative control. (**e**,**f**) The diagram shows L-plastin promoter–reporter constructs used in transfection assays indicating the putative AP4 site in the proximal promoter; relative luciferase activity was calculated as firefly luciferase activity compared to the pRL-TK Renilla transfection control plasmid. Three independent experiments were performed. Error bars indicated S.D.s (*n*=3), *P<0.05. **P<0.01

Given that promoter activity was reduced for the constructs with −216/+118 deletions, we attempted to identify the DNA-binding motif of the L-plastin promoter using TRANSFAC gene tool software. The results showed that oligonucleotide 1 has high sequence homology with putative AP4 binding sites (91%), oligonucleotide 6 has sequence homology with putative Aml-1 (85%) and SP1 (71%) binding sites, and oligonucleotides 8 and 10 have sequence homology with putative SRY (80%) and NKX3.1 (83%) binding sites. Moreover, the addition of an anti-AP4 antibody to nuclear extracts (NEs) of LNCaP and LNCaP-AI cells produced supershift complexes (*lane* 5 and *lane* 7, Figure 2a). The band intensity when using the NE from LNCaP-AI cells (*lane* 5) was much higher than that when using the NE from LNCaP cells (*lane* 7). A shifted band was noted in *lane* 3, which contained a mixture of the PC-3 cell NE and a standard AP4 oligonucleotide probe (Figure 2b). We subsequently performed chromatin immunoprecipitation (ChIP) analysis and observed that the level at which AP4 bound to the L-plastin promoter was statistically significant in LNCaP-AI cells, as shown in Figure 2c. Similarly, we found that AP4 bound directly to the L-plastin promoter in PC-3 cells (Figure 2d). The results of a relative luciferase activity assay support our hypothesis that transfection of the AP4-mutation (AP4-mut) plasmid consistently resulted in lower AP4-induced luciferase expression (nearly 40% lower) than transfection of the pGL3-0.2 plasmid in LNCaP cells stimulated with or without DHT (Figures 2e and f). Taken together, these findings indicate that AP4 binds to the L-plastin promoter in PCa cells, suggesting that AP4 may be important for regulating L-plastin promoter activity.

### Functional role of AP4 in L-plastin expression

**Figure 3:**
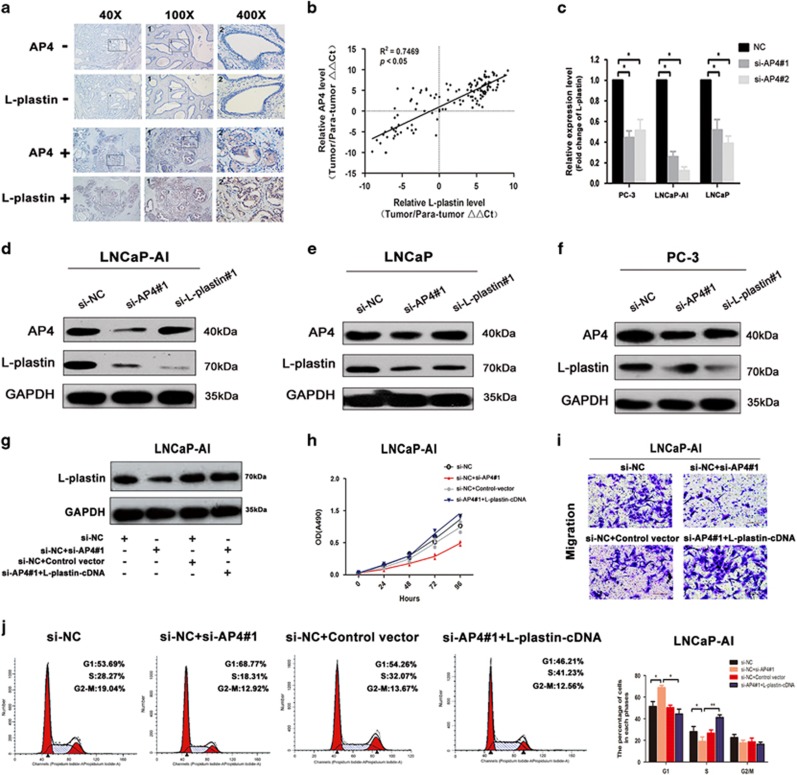
AP4 regulated PCa cells though upregulating L-plastin expression. (**a**) Representative IHC images of AP4 and L-plastin staining in the human PCa tissues. The micrographs at higher magnification showed weak immunostaining in both AP4 and L-plastin expressions in Case#1, while Case#2 showed strong immunostaining. (**b**) The association between AP4 and L-plastin mRNA levels in 136 PCa tissues (*R*^2^=0.7469, *P*<0.01). (**c**) The L-plastin expression after transfection with si-AP4#1 and si-AP4#2 was confirmed by qRT-PCR. (**d**–**f**) L-plastin expression was confirmed by western blot analysis after knocking down AP4 transcription using si-AP4#1 and L-plastin transcription using si-L-plastin#1 in LNCaP-AI, LNCaP and PC-3 cells. (**g**–**j**) AP4 knockdown was rescued proliferation, migration and invasion of the LNCaP-AI cells by the upregulation of L-plastin in western blotting analysis, MTT assay, transwell assays and cell cycle assays. Values represented the mean±S.D. from three independent experiments. *P<0.05, **P<0.01

To determine whether the transcription factor AP4 affects L-plastin expression in PCa cells, we down- and upregulated AP4 using small interfering RNAs (siRNAs) and AP4-pcDNA3.1 vectors (AP4-cDNA), respectively. We found that AP4 expression was significantly reduced by more than threefold by transfection with siRNA#1 and siRNA#2 compared with the negative controls (NCs) and that AP4 expression was significantly upregulated by transfection with AP4-cDNA compared with NC (Supplementary Figure S2). We then downregulated AP4 expression by si-AP4#2 and upregulated by AP4-cDNA and monitored the changes in mRNA levels by Affymetrix Microarray analysis (Supplementary Figure S3; GEO accession: GSE83140). The results demonstrated that L-plastin is one of differentially expressed downstream genes of AP4. Moreover, we examined whether the AP4/L-plastin axis observed in PCa cells is also present in clinical PCa samples. Based on immunohistochemistry (IHC) analysis, AP4 levels were positively associated with L-plastin levels in PCa specimens, as shown in Figure 3a. Moreover, this analysis revealed correlations between AP4 levels with L-plastin levels in PCa tissue samples from 136 patients (*R*^2^=0.7469, *P*<0.05, Figure 3b). In addition, qRT-PCR and western blotting showed that AP4 knockdown induced a decrease in L-plastin mRNA and protein expression in LNCaP-AI cells, whereas transfection with NCs did not alter L-plastin expression (Figures 3c–f). Taken together, these results demonstrate that AP4 possibly exerts its oncogenic effects in PCa cells by upregulating L-plastin.

### AP4 is regulated by the PI3K/AKT pathway to contribute to PCa progression

**Figure 4:**
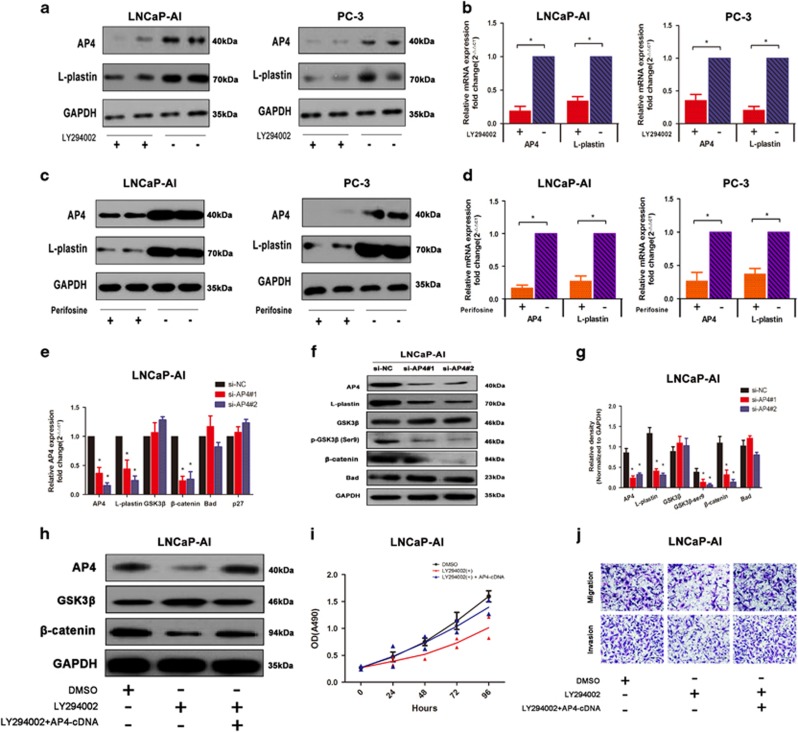
AP4 regulates PCa via activation of PI3K/AKT pathway. (**a**,**b**) LNCaP-AI and PC-3 cells were treated with PI3K inhibitor LY294002, AP4 and L-plastin mRNA and protein levels were determined by qRT-PCR and western blot analysis. GAPDH was used as loading control. (**c**,**d**) LNCaP-AI and PC-3 cells were treated with AKT inhibitor Perifosine, AP4 and L-plastin mRNA and protein levels were determined by qRT-PCR and western blot analysis. (**e**–**f**) The expressions of AP4, L-plastin, GSK3*β*, *β*-catenin and Bad after transfection with si-NC, si-AP4#1 and si-AP4#2 were examined by qRT-PCR and western blot analyses in LNCaP-AI cells. (**g**) Relative density of AP4, L-plastin, GSK3*β*, *β*-catenin and Bad protein expressions after normalization to GAPDH in LNCaP-AI cells. (**h**–**j**) The levels of AP4, *β*-catenin and GSK3*β* were examined with the PI3K inhibitor LY294002, and overexpression of AP4 could partly rescue the inhibitory effects in the changes of AP4 and *β*-catenin in western blotting analysis, MTT assay and transwell assays. Error bars indicate S.D.s (*n*=3), *P<0.05. **P<0.01

The PI3K/AKT pathway is frequently activated in PCa and has been demonstrated to play important roles in CRPC progression.^25, 26^ Accordingly, we evaluated the levels of AP4 and L-plastin after inhibition of PI3K/AKT pathway by qRT-PCR and western blotting, respectively. Inhibition of PI3K activity with LY294002 significantly downregulated AP4 and L-plastin expression levels (Figures 4a and b) and the inhibition of AKT by perifosine attenuated the AP4 and L-plastin expression levels (Figures 4c and d). These data revealed that AP4/L-plastin axis is regulated by PI3K/AKT pathway. Interestingly, microarray analysis showed that AP4 might exert its effects on several genes downstream of the PI3K/AKT pathway (Supplementary Figure S3). Moreover, western blotting was performed to show that the levels of phospho-GSK3*β* (ser9) and *β*-catenin in LNCaP-AI cells were significantly reduced in the AP4-knockdown group compared with the NC group (Figures 4e−g). GSK-3*β* activity is reduced by phosphorylation at Ser-9 leading to stabilization of *β*-catenin.^27^ In the present study, we found that the levels of GSK3*β* phosphorylation and *β*-catenin were decreased in the AP4-knockdown cells, indicating that AP4 promotes the activation of downstream PI3K/AKT pathway. Furthermore, in rescue experiments, the PI3K inhibitor LY294002 reduced AP4 and *β*-catenin levels, and AP4 overexpression partially rescued the inhibitory effects of LY294002 on AP4 and *β*-catenin expression (Figure 4h). MTT and transwell assays were used to demonstrate that inhibition of PI3K rescued LNCaP-AI cell proliferation, migration and invasion by AP4 overexpression (Figures 4i and j). Taken together, these data indicate that the AP4/L-plastin axis is regulated by the PI3K/AKT pathway, which contributes to PCa metastasis and castration resistance.

### AP4 increases CRPC cell proliferation, migration and invasion *in vitro*

**Figure 5:**
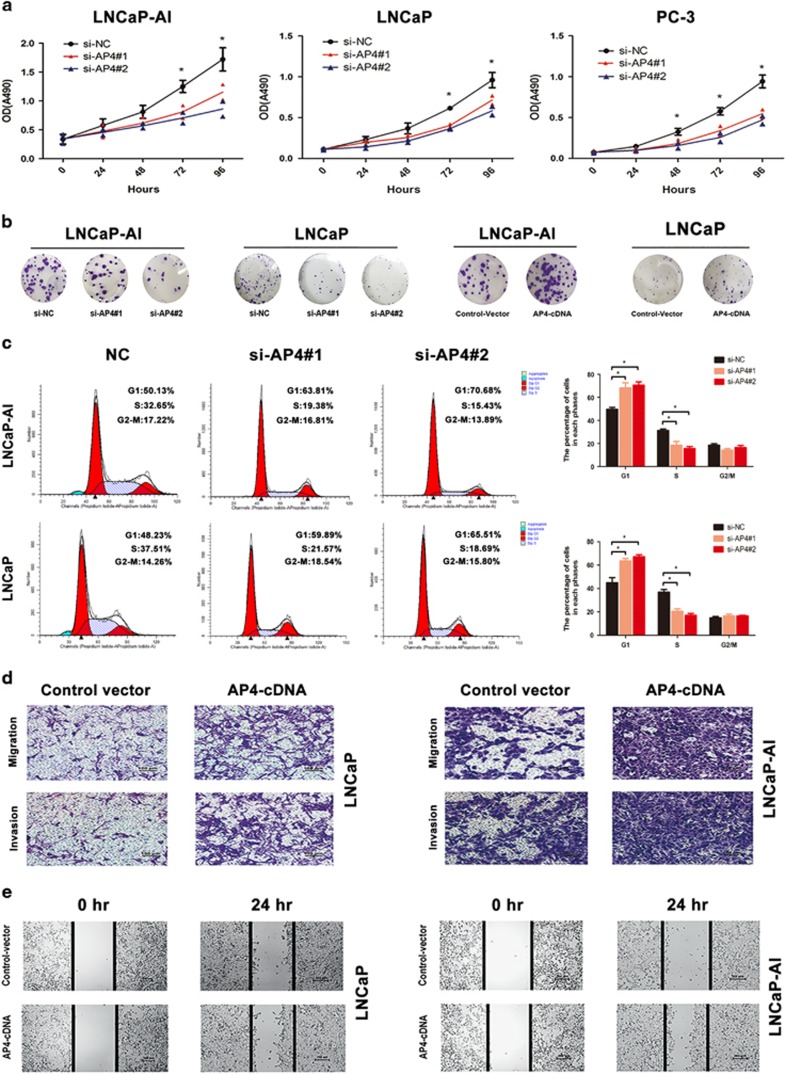
AP4 increases the proliferation, migration and invasion of PCa cells *in vitro*. (**a**) AP4 knockdown decreased the proliferation as determined by MTT assay. (**b**) Effect of AP4 knockdown or overexpression on colony formation was measured in LNCaP and LNCaP-AI cells. Representative images of the colonies, stained with crystal violet. (**c**) LNCaP and LNCaP-AI were transfected with si-AP4#1 or si-AP4#2 and analyzed by flow cytometry. Percentages of cell populations at different stages of cell cycles are listed within the panels. (**d**) The motility of PCa cells transfected with AP4-cDNA when compared with the controls by the wound healing assay. (**e**) The migration and invasion of PCa cells were transfected with AP4-cDNA compared with NC by transwell assays. Values represented the mean±S.D. from three independent experiments. *P<0.05, **P<0.01

Downregulation of AP4 expression resulted in decreased tumour cell proliferation in the LNCaP-AI, LNCaP and PC-3 cell lines, as demonstrated by MTT and colony formation assays; conversely, overexpression of AP4 had the opposite effects on proliferation in these cell lines (Figures 5a and b). In addition, flow cytometry assays demonstrated that compared with the NC group, AP4 knockdown significantly increased the population of cells in G1 phase, whereas it reduced the population in S phase (Figure 5c). Transwell assays showed that AP4 overexpression significantly increased LNCaP-AI and LNCaP cell migration and invasion (Figure 5d), whereas AP4 knockdown had the opposite effects (Supplementary Figure S4). The results of wound healing assays were similar to those of the transwell assays (Figure 5e).

Moreover, we performed rescue experiments to determine whether AP4-regulated L-plastin expression contributes to PCa progression. Western blot analysis showed that AP4 knockdown in LNCaP-AI cells reduced the level of L-plastin expression and that overexpression of L-plastin was able to partially reverse these effects (Figure 3g). Using MTT and transwell assay, we also found that simultaneously L-plastin overexpression rescued the inhibition of proliferation and migration in AP4 knockdown cells, respectively (Figures 3h and i). Furthermore, cell cycle assays showed that transfection of L-plastin-cDNA into AP4-knockdown cells could partially downregulate the increase in the G1-phase population (Figure 3j). We also co-transfected cells with AP4-cDNA and si-L-plastin#3 and found that the L-plastin knockdown could attenuate the increases in cell proliferation induced in AP4-cDNA cells by MTT and cell cycle assays (Supplementary Figure S5). Altogether, these results demonstrate that L-plastin is an important downstream molecule in AP4-mediated regulation of proliferation, migration and invasion in PCa cells.

### AP4 promotes PCa tumourigenicity and metastatic potential *in vivo*

**Figure 6:**
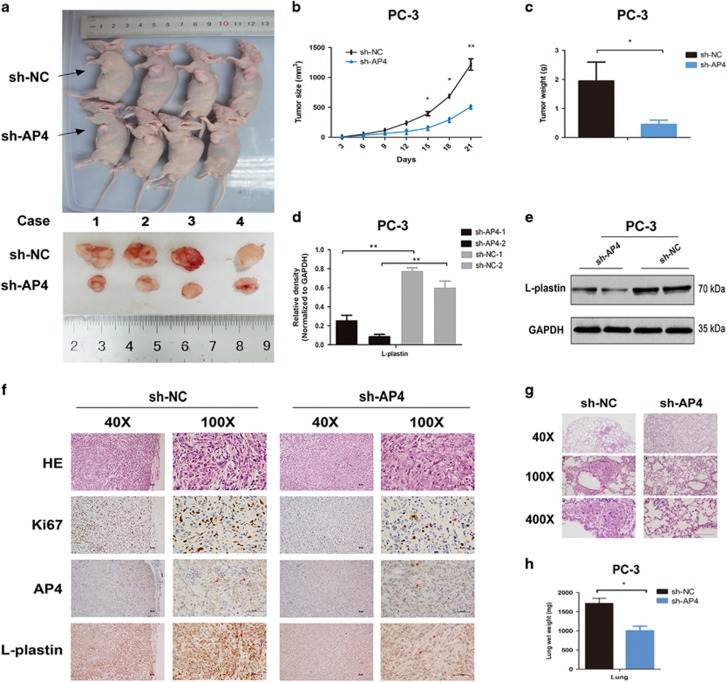
AP4 promotes tumorigenicity and metastatic potential *in vivo*. (**a**–**c**) The images of animals and tumors were shown. Tumor weights were shown as the means±S.D. when the tumors were collected. (**d**,**e**) qRT-PCR and western blot analyzed the expression of L-plastin in tumor tissues from sh-AP4 in PC-3 cells compared with sh-NC cells. (**f**) Representative images of HE and IHC staining of the tumor. The IHC staining showed that AP4 knockdown decreased the proliferation index Ki67. (**g**,**h**) Representative images of lung metastasis of subcutaneous xenografts assay. Histological analysis of lung wet weight is presented as the mean±S.D. (*n*=4). *P<0.05, **P<0.01

Consistent with the results of our *in vitro* experiments, the results of *in vivo* experiments showed that tumour growth in the AP4 shRNA (sh-AP4) group was significantly decreased compared with the NC group (Figure 6a). Both tumour size and tumour weight were significantly decreased in the sh-AP4 group compared with the NC group at 3 weeks after injection (Figures 6b and c). Inhibiting AP4 expression in the xenotransplanted tumours resulted in lower L-plastin expression as demonstrated by qRT-PCR and western blotting (Figures 6d and e). We also found that tumours from the sh-AP4 group exhibited a decreased rate of Ki-67 positivity and less intense IHC staining compared with tumours from the NC group (Figure 6f). To validate the effects of AP4 knockdown on PCa metastatic potential *in vivo*, we induced metastasis in mice that had received subcutaneous xenografts and tail vein xenografts and then compared the lung wet weights and average numbers of metastatic foci in these mice with those in control mice. The results for the subcutaneous xenograft tumour model indicated that AP4 not only promoted local tumour growth in nude mice but also significantly potentiated the metastasis of tumour cells to the lung (Figures 6g and h). In addition, we noted reduced lung colonization and lower lung wet weights in the AP4-knockdown group than in the NC group in the tail vein metastasis model (Supplementary Figure S6). Taken together, these results demonstrate that AP4 plays a crucial role in tumourigenicity and metastasis *in vivo*.

### AP4 and L-plastin upregulation is correlated with a poor prognosis in human CRPC

**Figure 7:**
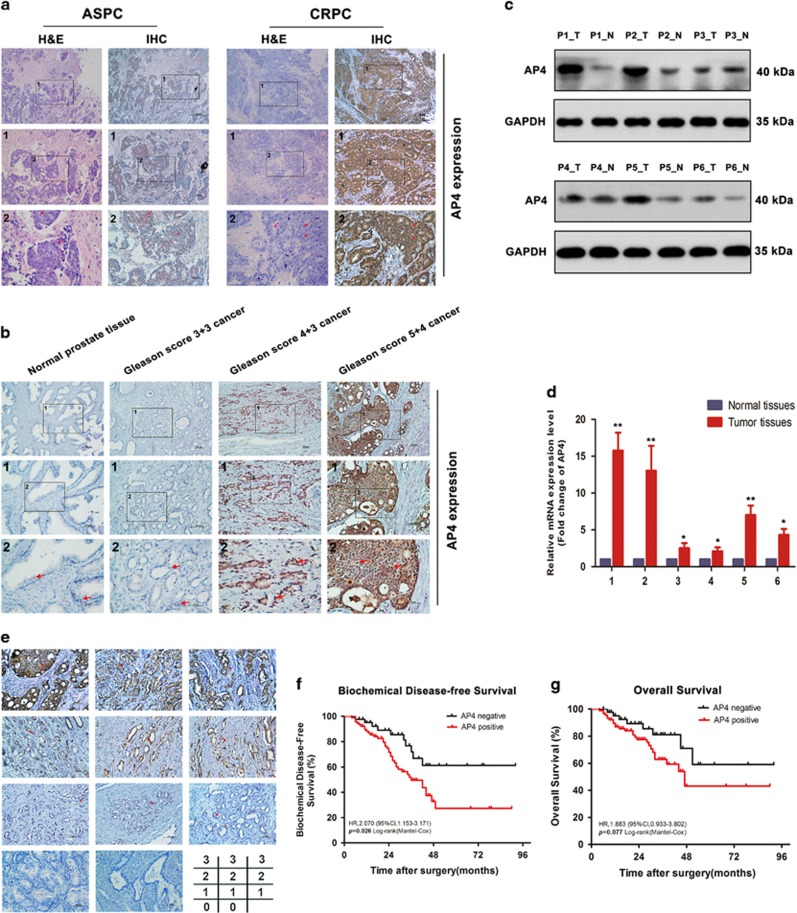
Upregulation of AP4 correlated with poor prognosis in human CRPC. (**a**) Representative images of H&E and IHC in androgen-sensitive PCa (ASPC) and castration-resistant PCa (CRPC) (*n*=8) with different AP4 staining. (**b**) Representative images of AP4 staining in normal tissue, Gleason score 3+3, Gleason score 4+3 and Gleason score 5+4, respectively. (**c**,**d**) Western blot analyses and qRT-PCR of AP4 expression in PCa tissues (T) and matched adjacent non-tumour tissues (N). The mRNA and protein levels were normalized to GAPDH. (**e**) Representative images of AP4 staining in human PCa tissues (score 0–score 3). (**f**,**g**) Kaplan–Meier survival curves for bDFS and OS of PCa patients (*n*=133) with different AP4 staining scores (the score of 0 or 1 represented AP4 negative, while 2 or 3 represented AP4 positive)

To determine the clinical significance and biological role of AP4 in human CRPC, we detected AP4 expression in human PCa tissues and found that AP4 expression was increased in PCa tissues (*n*=136) and CRPC tissues (*n*=8) collected from patients with urinary obstruction compared with paired normal prostate tissues (*n*=136) and paired androgen-sensitive PCa (ASPC) tissues (*n*=8) (Figures 7a and b,Table 1 and Supplementary Table S3). AP4 was also significantly overexpressed at the protein and mRNA levels in PCa tissues compared with matched adjacent non-tumour tissues (Figures 7c and d). Statistical analysis revealed that AP4 expression levels were positively correlated with Gleason scores over 7 (*P*<0.05) and lymph-node metastasis (*P*<0.001) (Table 1). Importantly, we found that higher AP4 expression levels were correlated with shorter biochemical disease-free survival (bDFS) in patients with PCa than lower AP4 expression levels (HR, 2.070, 95% CI, 1.153-3.171; *P*<0.05). However, the AP4-positive group did not differ significantly from the AP4-negative group with respect to overall survival (OS) (HR, 1.883, 95% CI, 0.933-3.802; *P*=0.077) (Figures 7e–g). Univariate and multivariate Cox regression analyses showed that AP4 expression was an independent prognostic factor for survival in patients with PCa (*P*<0.05; Supplementary Table S2).

We also found that L-plastin was overexpressed in PCa tissues (*n*=136) compared with paired normal prostate tissues (*n*=136) and that L-plastin levels were positively associated with pathological stages higher than T2 (*P*<0.05) and pathological lymph-node metastasis (*P*<0.05) (Supplementary Figure S7 and Table 1). The Oncomine data showed that L-plastin expression levels were also upregulated in patients with PCa^28, 29, 30, 31^ compared with patients with normal prostate glands and patients with metastatic PCa^32^ compared with patients with primary PCa (Supplementary Figure S8).

## Discussion

CRPC is the major cause of mortality from PCa and the current studies on CRPC development have predominantly focused on androgen metabolism and androgen receptor pathways. ^38^ Identifying other molecular phenomena associated with CRPC may enable us to control this common and lethal male malignancy. Previous studies have shown that the PI3K/AKT signalling pathway plays a key role in CRPC development and maintenance^10, 11, 33^ and PI3K/AKT signalling pathway deregulation occurs in almost all cases of advanced CRPC. In the present study, we demonstrated that AP4 upregulated L-plastin by binding its promoter and AP4 is regulated by the PI3K/AKT signalling pathway to sustain PCa cell growth under androgen deprivation-induced stress and lead to CRPC. This mechanism in which AP4 is regulated by the PI3K/AKT signalling pathway to promote PCa progression is crucial for understanding the mechanics of existing therapies and may support the development of PI3K/AKT combination therapies for PCa patients.

We previously reported that L-plastin expression is upregulated by androgen and oestrogen in LNCaP cells, possibly through the AREs and EREs in its promoter.^16^ We also recently found that L-plastin expression is consistently activated even after androgen deprivation, suggesting that L-plastin plays crucial role in CRPC cell growth. Here, to determine whether L-plastin functions in PCa progression, we established an androgen-independent LNCaP subline known as LNCaP-AI. Moreover, we found that the binding of AP4 to the L-plastin promoter may be the key androgen-independent event that underlies CRPC development. These data indicated that AP4 upregulated L-plastin by binding to its promoter, leading to PCa progression.

In addition to a role in the development of CRPC, AP4 was found to be associated with other types of cancer, including colorectal cancer,^17^ hepatocellular cancer,^34^ gastric cancer^35, 36^ and lung cancer.^37^ The transcription factor AP4 was first identified as a cellular factor that activated late SV40 transcription *in vitro* by synergistically interacting with AP-1.^38^ Large amounts of evidence support the hypothesis that AP4 preferentially binds to the E-box motif CAGCTG to activate the expression of its downstream target genes. Imai *et al.* showed that AP4 interacted with histone-modifying enzymes, such as HDAC1 and HDAC3, to repress HIV1 expression and PAXH-AP1 gene expression,^39^ and Jackstadt *et al.* demonstrated that AP4 is encoded by a c-MYC target gene and is upregulated along with c-MYC in colorectal cancer.^20^ These findings supported our hypothesis that AP4 regulates CRPC development by directly binding to the L-plastin promoter, which increases L-plastin expression, and our results serve as evidence supporting the notion that AP4 is functionally and clinically associated with PCa proliferation, migration and invasion. Our findings also serve as a basis for the performance of additional studies regarding the mechanisms through which AP4 regulates L-plastin expression in PCa development and progression. Identifying the probable mechanisms through which AP4 exerts its effects in PCa may facilitate the development of targeted therapies to treat CRPC.

The PI3K/AKT signalling pathway is elevated in a significant portion of primary and metastatic PCa.^26, 40^ In this study, we demonstrated the AP4 is the crucial component of PI3K/AKT pathway in PCa. Our findings showed that AP4/L-plastin axis is regulated by PI3K/AKT pathway. Interestingly, we also found that AP4 could exert its effects on several PI3K/AKT pathway downstream genes by Microarray analysis. In addition, western blot analysis indicated that the levels of phospho-GSK3*β* (ser9) and *β*-catenin are decreased in the AP4-knockdown cells. As we know, GSK3*β*, a serine/threonine kinase, is an important downstream target of PI3K/AKT pathway^41, 42^ and the activity of GSK-3*β* is reduced by phosphorylation at Ser-9.^27^ Thus, the levels of GSK3*β* phosphorylation and *β*-catenin are decreased in the AP4-knockdown cells, indicating that AP4 could promote the activation of PI3K/AKT pathway. These results suggest that AP4 might be an essential element in a positive feedback loop sustaining activation of PI3K/AKT pathway in PCa. It is our hope that these findings will provide researchers with new ideas leading to the development of therapies capable of treating cancer progression and invasion by targeting the transcription factor AP4.

In summary, we demonstrated that the transcription factor AP4 upregulated L-plastin by binding to its promoter and that AP4 is regulated by the PI3K/AKT signalling pathway to induce PCa cell proliferation, migration and invasion. We also showed that AP4 is overexpressed in PCa tissues and is an independent prognostic factor in patients with PCa. Therefore, understanding the precise regulatory mechanism of AP4 in PCa progression will not only advance our knowledge of the tumourigenesis of PCa but will also permit the development of novel therapeutic strategies and help identify an effective biomarker to predict outcomes for PCa patients.

## Materials and methods

### Patients and clinical samples

A total of 136 paraffin-embedded prostate carcinoma specimens were obtained from patients undergoing radical prostatectomy at Sun Yat-Sen Memorial Hospital between February 2005 and July 2015. CRPC was diagnosed in accordance with European Association of Urology Guidelines.^1^ Written informed consent was obtained from all patients before sample collection, and the use of human samples for this study was approved by the appropriate hospital ethics review committees. Detailed clinicopathologic characteristics of the enrolled patients are summarized in Table 1.

### Cell lines and cell culture

The prostate carcinoma cell lines used in this study were obtained from American Type Culture Collection (ATCC, Manassas, VA, USA). All media in which the cells were cultured were supplemented with 1% streptomycin/penicillin, and the cells were maintained at 37 °C in a humidified atmosphere with 5% CO_2_. Additional information regarding the procedures is presented in Supplementary Methods.

### L-plastin promoter constructs

The promoter sequence of L-plastin was identified and reported (GenBank # : AH002870), and promoter constructs containing the region from −2197 nt to +118 nt relative to the putative transcription start site were amplified from human genomic DNA by PCR using specially designed primers containing *Xho*I and *Bgl*II restriction sites. These experiments were conducted as previously described.^21^

### PCR-based site-directed deletion

A DNA fragment containing a steroid receptor binding site was amplified by PCR using specially designed primers (Supplementary Table S1) and subsequently used to introduce specific base pair substitutions into other DNA sequences with an ExSiteTM PCR-based Site-directed Mutagenesis Kit (Stratagene, La Jolla, CA, USA) according to the manufacturer’s instructions.

### Luciferase activity assay

LNCaP, LNCaP-AI and PC-3 cells were seeded in six-well plates at a density of 1 × 10^5^ per well and cultured in 1 ml of complete medium. The cells were incubated overnight before being transiently transfected using Lipofectamine RNAi Max (Life Technologies, Carlsbad, CA, USA) with 1 *μ*g each of mutant L-plastin promoter constructs produced by site-deleted and nest-deleted mutagenesis.

### Chromatin immunoprecipitation

ChIP was conducted with an EZ-Magna ChIP A/G Kit (Millipore, Bedford, MA, USA) according to the manufacturer’s instructions. ChIP-qRT-PCR was conducted as previously described.^21^

### Electrophoretic mobility shift assay and the supershift assay

NEs were prepared using a nuclear extraction kit (Active Motif, Carlsbad, CA, USA) according to the manufacturer’s instructions. EMSA and the supershift assay were conducted as previously described.^21^

### Microarray analysis

In this study, microarray analysis was performed by Gene Tech Corporation (Shanghai, China) using PrimeViewTM Human Gene Expression Array (Affymetrix) according to the manufacturer’s instructions. All primary data pertaining to the microarray analysis are available at the Gene Expression Omnibus (GEO accession: GSE83140) website.

### Cell transfection and viral infection

LNCaP-AI, LNCaP and PC-3 cells were transfected with siRNA using Lipofectamine RNAi Max (Life Technologies) according to the manufacturer’s instructions. The sequences of the siRNAs used herein are provided in Supplementary Table S1. For rescue experiments, we transfected the above cells with si-NC and si-AP4#2 for 24 h, after which we introduced the L-plastin-cDNA or a control vector into the cells for 36 h before collecting them.

### Tumourigenesis study in a nude mouse model

An *in vivo* tumourigenesis study was performed as described in Supplementary Methods. Nude mice (4–6 weeks old, 18–20 g, Vital River Laboratories, Beijing, China) were used and maintained in a specific pathogen-free environment at the Laboratory Animal Center of Sun Yat-Sen University. AP4-knockdown or NC-transfected PC-3 cells (5 × 10^6^) were suspended in 200 *μ*l of phosphate-buffered saline (PBS) and subcutaneously injected into the right or left side of the dorsum of five mice. The mice were killed at 21 days post-injection, and the tumours were collected for further study. To establish the subcutaneous xenograft tumour model, we injected a total of 1 × 10^7^ transfected cells into the left side of the dorsum of 6-week-old nude mice. After 6 weeks, these animals were killed, and the lungs were removed and weighed. For metastasis experiments, we injected 1 × 10^7^ transfected cells into the tail veins of nude mice, which were killed 6 weeks later. Tumour burdens were examined with a microscope, and the total number of metastatic foci on the surface of each lung was counted.

### Cell proliferation, colony formation assay and cell cycle analysis

Methyl thiazolyl tetrazolium (Promega, Madison, WI, USA) colourimetric assay was used to assess cell viability. For the colony-formation assay, we seeded transfected cells in six-well plates and maintained them in F12K medium containing 10% fetal bovine serum for 2 weeks. The colonies were then fixed with methanol, stained with 0.1% crystal violet (Sigma-Aldrich, Milwaukee, WI, USA) and counted. For cell cycle analysis, cells were collected at 48 h after transfection and fixed in 70% ice-cold ethanol before being treated with RNase A and stained with 50 mg/ml propidium iodide for DNA content analysis, which was performed with a FACSCalibur BD Flow Cytometer. The data were collected and processed using BD FACSuite analysis software.

### Protein extraction and western blot analysis

Western blotting was performed as described in Supplementary Methods. Antibodies against the following proteins were used for this experiment: AP4 (ab28512, 1 : 1000, Santa Cruz Biotechnology, Santa Cruz, CA, USA), L-plastin (ab109129, 1 : 1000, Abcam, Shanghai, China), AR (ab9747, 1 : 200, Abcam), p27 (ab32034, 1 : 1000, Abcam), Bad (ab32445, 1 : 2500, Abcam), *β*-catenin (ab32572, 1 : 5000, Abcam), GSK3*β* (#5676, 1 : 1000, CST), GSK3*β* ser9 (#9322, 1 : 1000, CST) and glyceraldehyde-3-phosphate dehydrogenase (#5174, 1 : 1000, CST, Beverly, MA, USA).

### Immunohistochemical staining and scoring

Paraffin-embedded primary carcinoma specimens and xenograft tumour specimens from the abovementioned nude mice were stained for AP4 and then incubated overnight at 4 °C with a rabbit anti-AP4 antibody (ab28512, Abcam). After washing three times in PBS, the sections were immunostained with a donkey anti-rabbit secondary antibody (ab1500075, Abcam) for 1 h at 37 °C. Anti-AP4 and anti-Ki67 antibodies (1 : 1000, Zhongshan Bio-Tech Co. Ltd, Beijing, China) were used to detect AP4 and Ki67 expression in the nude mouse tumours. To evaluate and grade the AP4 staining results, we used a scoring system previously devised by Ohara *et al.*^43^ Briefly, AP4 staining intensity was graded on a scale of 0–3 (0, no staining; 1, weak staining; 2, moderate staining; and 3, strong staining) (Figure 7e).

### Bioinformatics analysis

Potential transcription factor binding sites in the human L-plastin promoter were analysed using TRANSFAC gene tool software (http://www.gene-regulation.com), with a cut-off value of 0.70. Data sets from the Oncomine cancer database (https://www.oncomine.com/resource/main.html) were used to determine L-plastin mRNA expression levels, which are expressed as fold changes in gene expression relative to a control. The statistical significance of these fold changes was determined by *P*-values.

### Statistical analysis

All quantitative data are presented as the mean±standard deviation from at least three independent experiments. Chi-square tests (*χ*^2^ tests) were used to assess relationships between non-parametric variables, and Student’s *t*-test or one-way analysis of variance was used to evaluate relationships between parametric variables (two-tailed). bDFS and OS were assessed using the Kaplan–Meier method. All statistical analyses were conducted using SPSS v.16.0 (SPSS Inc., Chicago, IL, USA), and *P*-values<0.05 were considered statistically significant.

## Figures and Tables

**Table 1:** Correlation between AP4 and L-plastin expression and clinicopathologic characteristics of PCa patients

**Characteristics**	**Patient frequency (%)**	**AP4 expression level**			L**-plastin expression level**		
		**Low**	**High**	***P*-value**^a^	**Low**	**High**	***P*-value**^a^
Total cases	136	44 (32.4%)	92 (67.6%)		38 (27.9%)	98 (72.1%)	
*Age (years)*				0.342			0.816
<65	45 (33.1%)	17	28		12	33	
⩾65	91 (66.9%)	27	64		26	65	
*PSA level (μg/l)*				0.185			0.278
⩽10	51 (37.5%)	20	31		17	34	
>10	85 (62.5%)	24	61		21	64	
*Gleason score*				0.040*			0.259
⩽7	115 (84.5%)	41	73		30	85	
>7	21 (15.5%)	3	19		8	13	
*Pathologic stage*				0.051			0.026*
⩽T2	65 (47.8%)	27	40		24	41	
>T2	71 (52.2%)	17	52		14	57	
*Lymph-node status*				0.001*			0.022*
Negative	99 (72.8%)	40	59		33	66	
Positive	37 (27.2%)	4	33		5	32	

a Chi-square test, *P<0.05, **P<0.01

Bold values indicate statistically significant values.
