# Supplementary material for: AP4 modulated by the PI3K/AKT pathway promotes prostate cancer proliferation and metastasis of prostate cancer via upregulating L-plastin
Source: Cell Death Dis. 2017 Oct 5;8(10):e3060–. doi: 10.1038/cddis.2017.437 (PMC5680569; doi:10.1038/cddis.2017.437)
Supplement: Supplementary Data [file cddis2017437x1.doc]

# Supplementary data

# Supplemental Figures


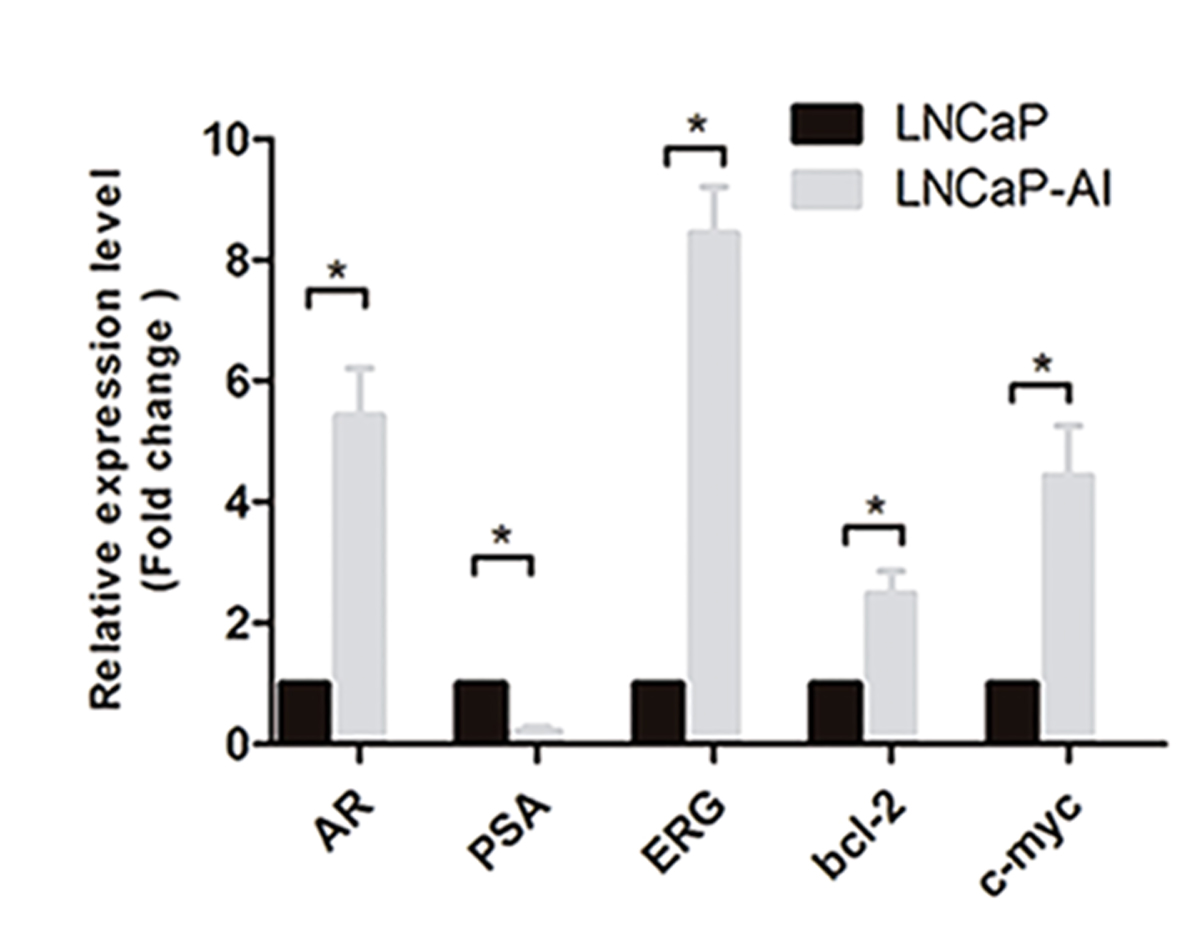


Figure S1. The expressions of AR, PSA, ERG, bcl-2 and c-myc were examined by qRT-PCR in LNCaP and LNCaP-AI cells.


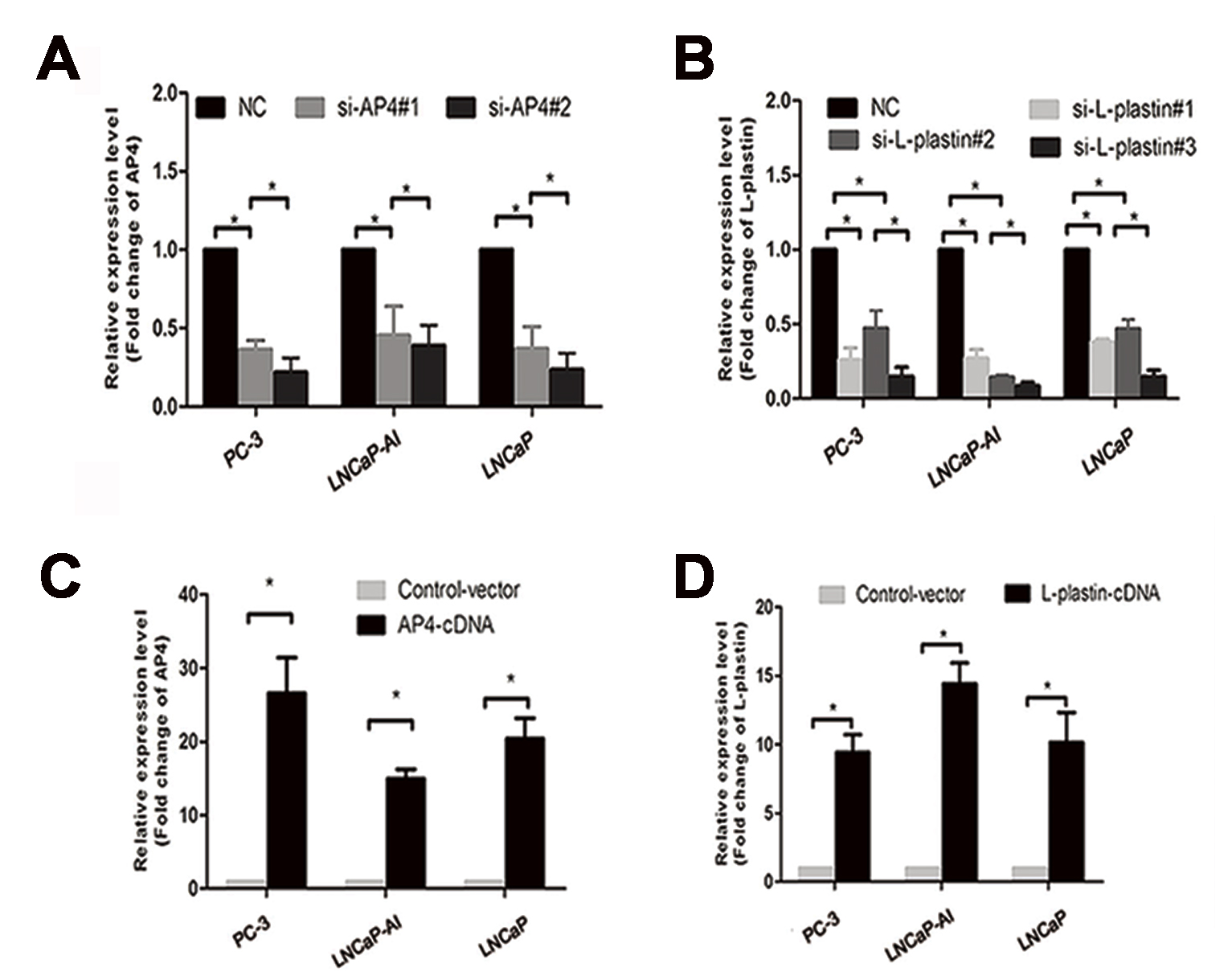


Figure S2. The AP4 and L-plastin expression levels after transfection with siRNAs (A-B) and cDNA (C-D) were confirmed by qPCR in PCa cell lines.


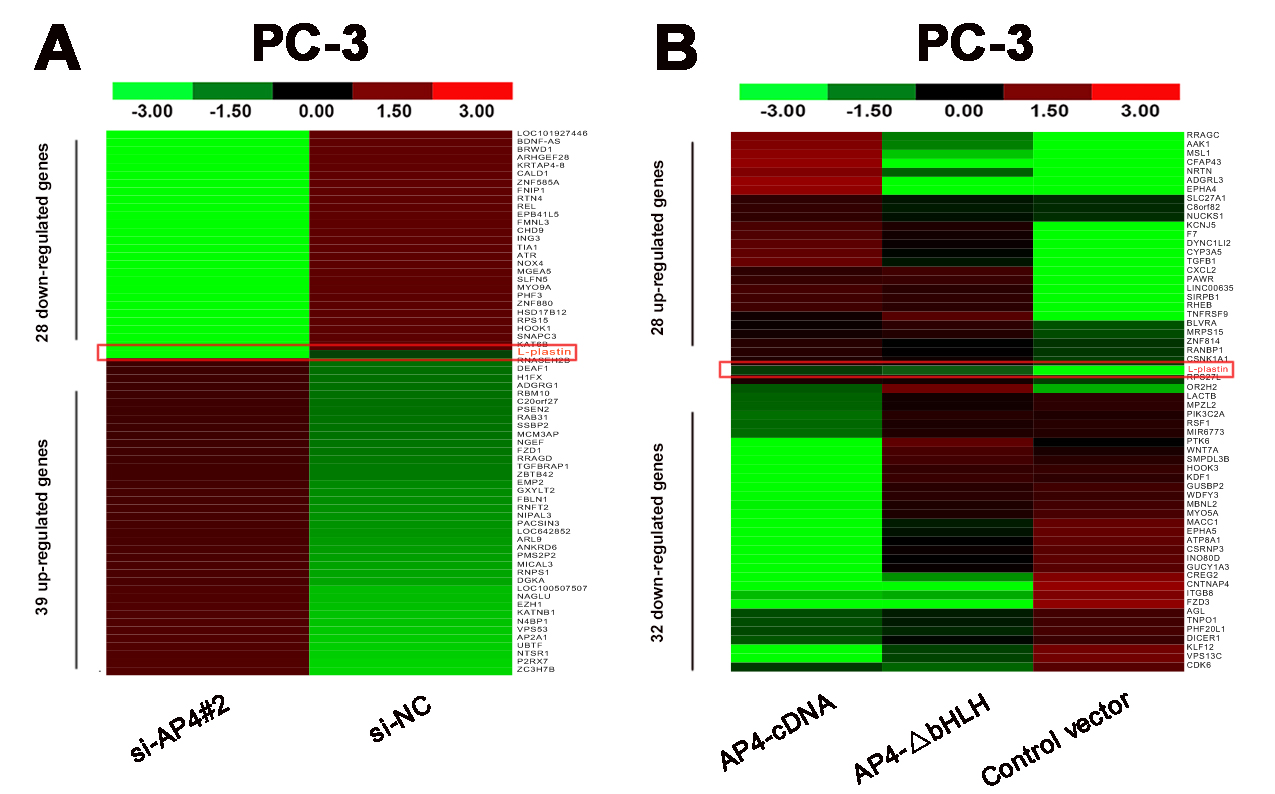


Figure S3. The AP4 expression was downregulated by si-AP4#2 and upregulated by AP4-cDNA and monitored the changes in mRNA levels by Affymetrix Microarray analysis.


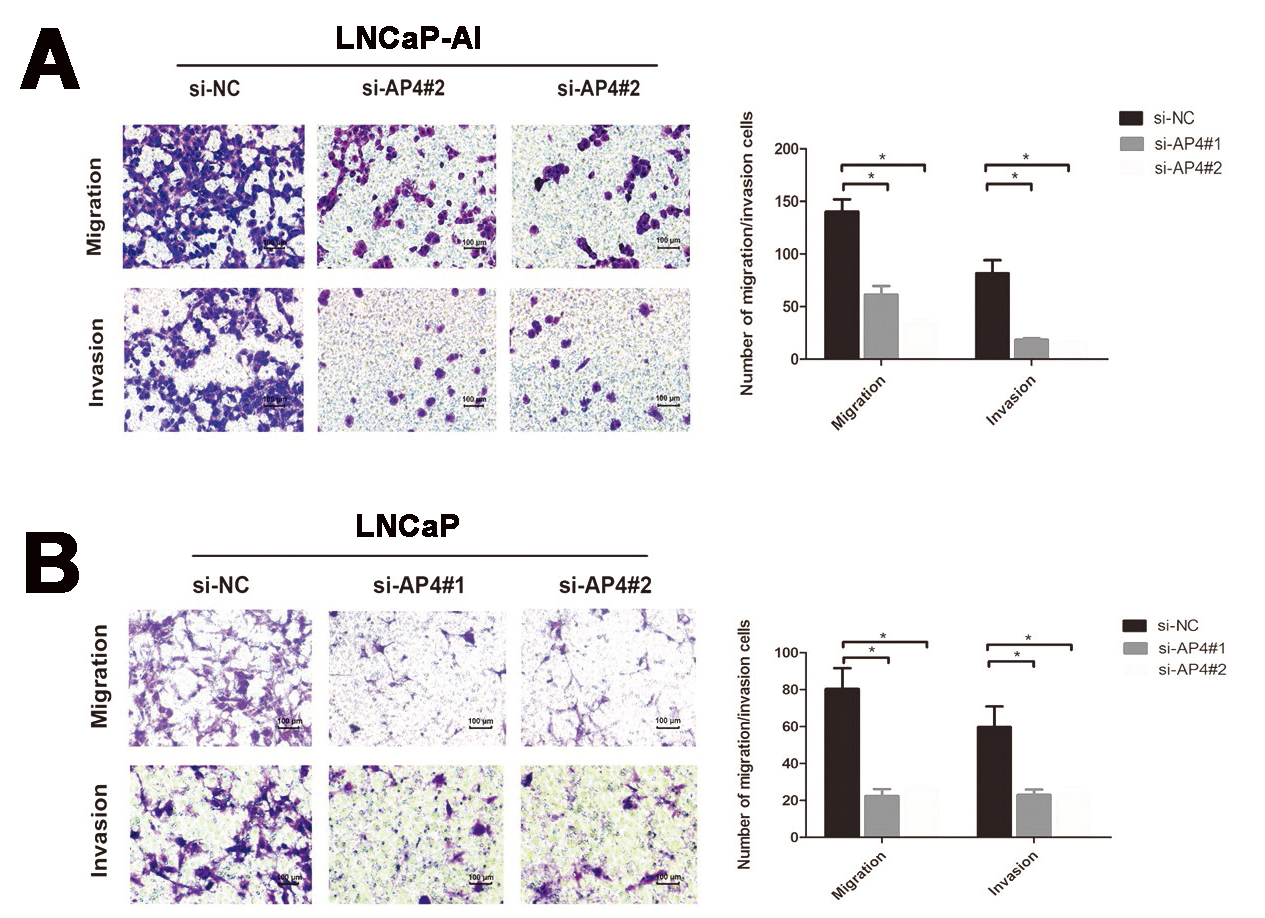


**Figure S4. The migration and invasion of LNCaP-AI cells (A) and LNCaP cells (B) transfected with si-AP4#1 and si-AP4#2 when compared with the negative control by transwell assays.** Values represented the mean ± SD from three independent experiments. **p* < 0.05, ***p* < 0.01.


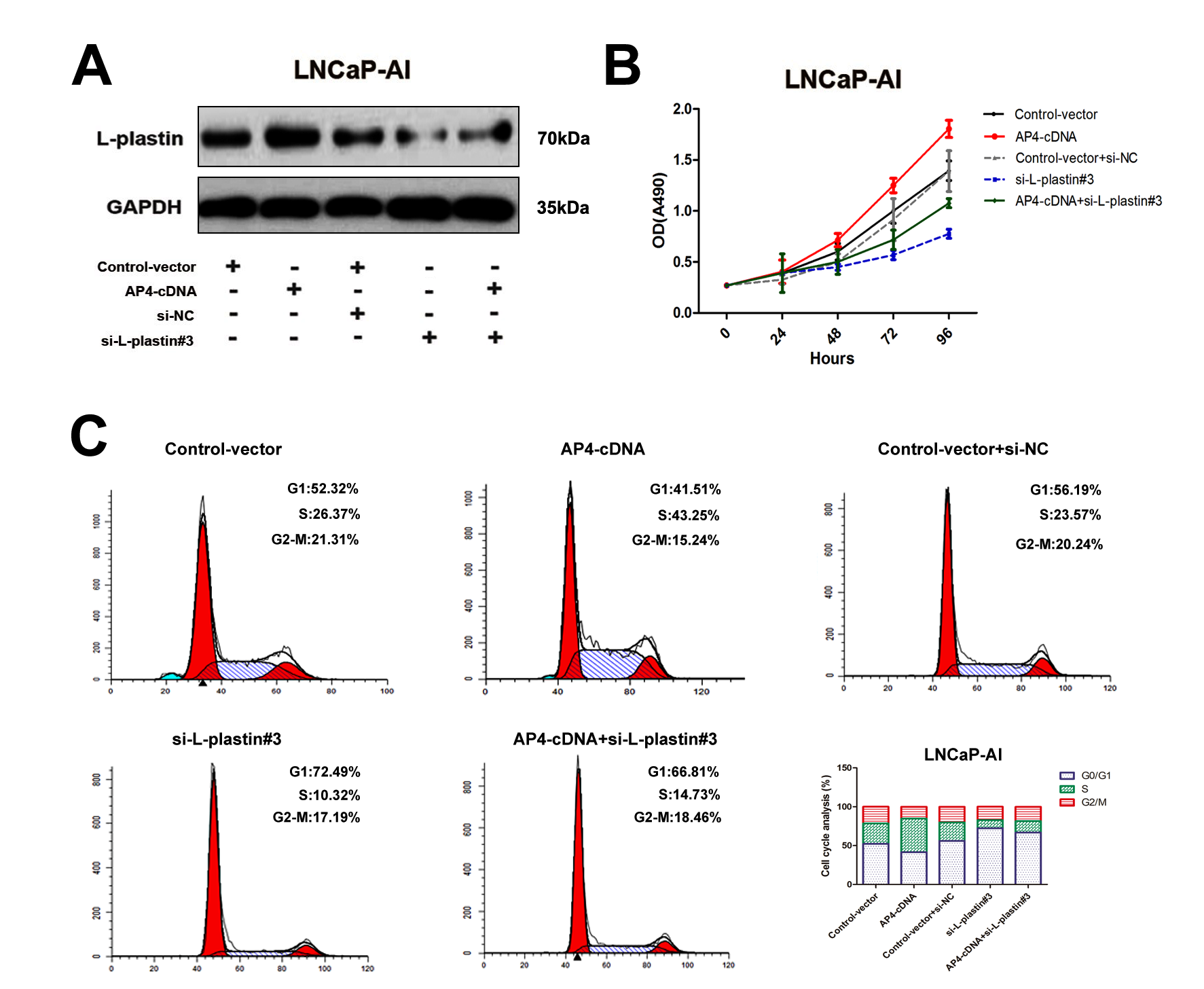


Figure S5. L-plastin was a major downstream gene of AP4**.** AP4 overexpression promoted LNCaP-AI cells proliferating can be attenuated by downregulation of L-plastin in western blotting analysis (A), MTT assay (B) and cell cycle assays (C).


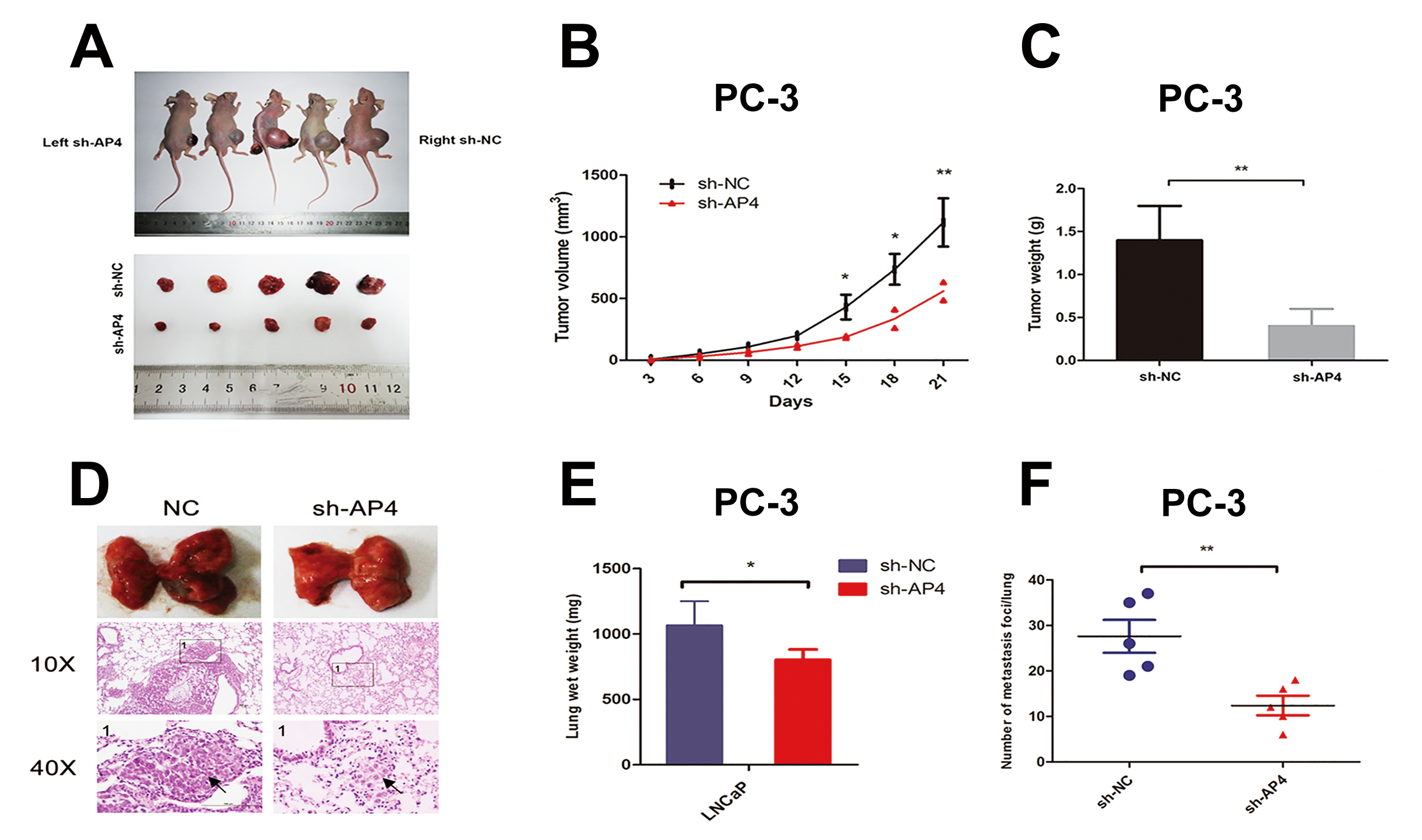


Figure S6 **AP4 promotes tumorigenicity and metastatic potential *in vivo*.** (A-C) The images of animals and tumors were shown (n = 5). Tumor weights were shown as the means ± SD when the tumors were harvested. (D-F) Representative images of lung metastasis of tail vein assay. Histological analysis of lung wet weight and the average number of foci is presented as the Mean ± SD (n=5). **p* < 0.05, ***p* < 0.01.


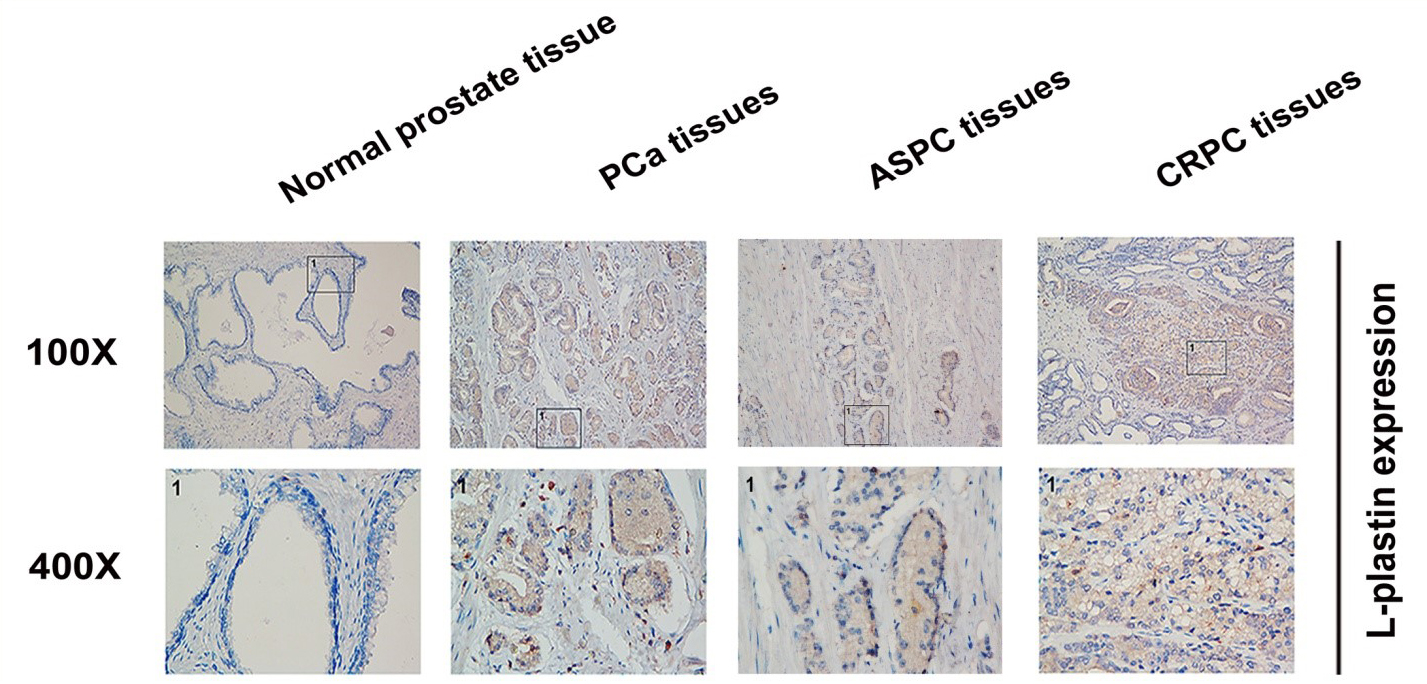


Figure S7 Representative IHC images of L-plastin staining in human normal prostate tissues and PCa tissues (n =136), and ASPC tissues and CRPC (n=8).


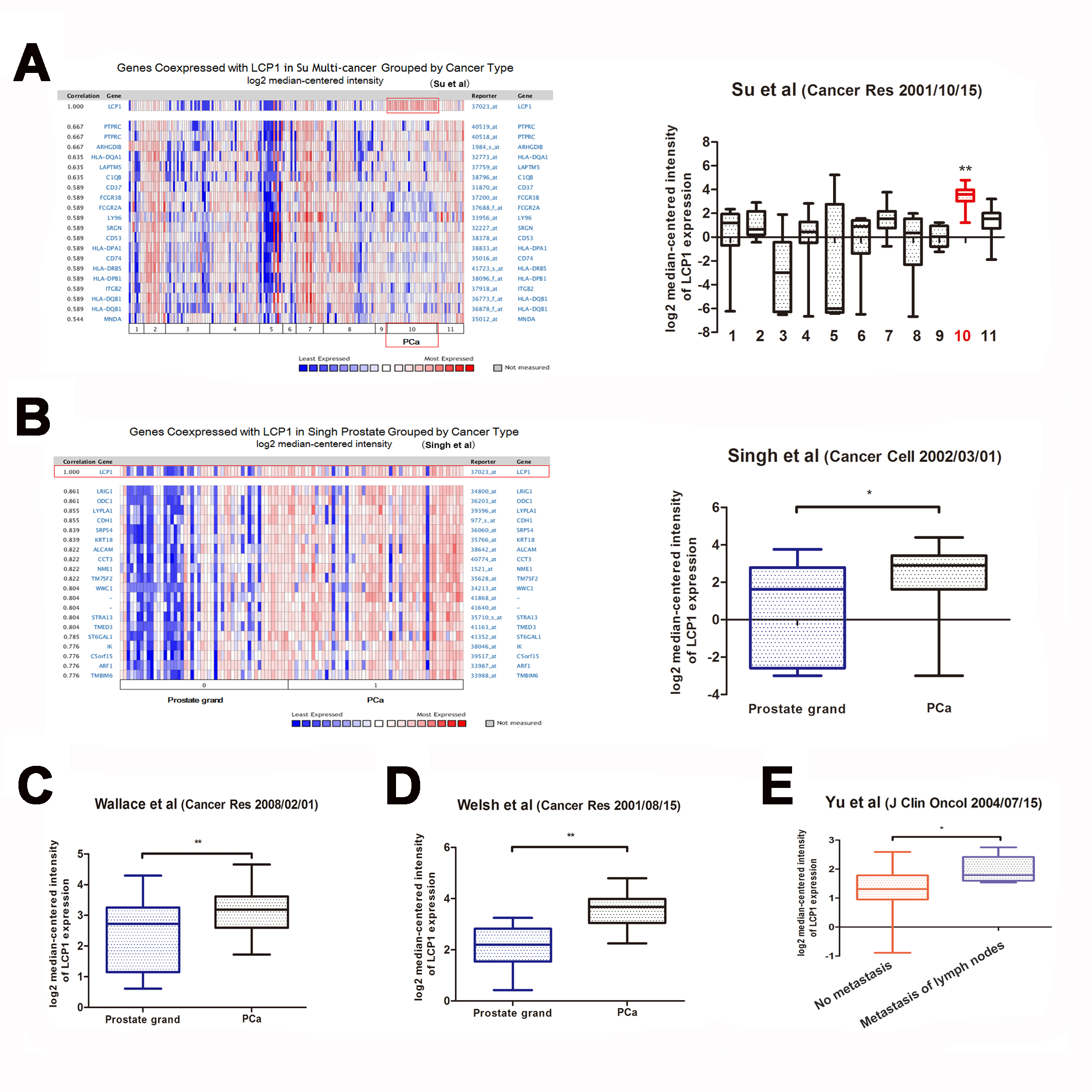


**Figure S8. The mRNA levels of L-plastin (LCP1) in PCa tissues is higher than normal prostate tissues.** Selected datasets from the Oncomine cancer microarray database determined the alterations of L-plastin in mRNA expression levels. Student’s t tests were performed for statistical significance for the entire series of analysis.

| Supplementary Tables **Table S1 Primer of experiments** | | |
| --- | --- | --- |
| **Primer name** | **Nucleotide Sequence (5’-3’)** | **Application** |
| L-plastin_promoter_F-XhoI | CGAT**CTCGAG** GTACCCAGGTCAATGCCTTTCATCT | L-plastin Promoter Constructs |
| L-plastin_promoter_R-HindIII | GCAT**AAGCTT** CATTAGCCAGTGGTGTGGTGACT | — |
| L-plastin F | ACAATTGCCTGAAACTGGGT | PCR |
| L-plastin R | TTGTGATTTCCCAGGGAAGA | — |
| GAPDH F | GAAGGTGAAGGTCGGAGTC | qPCR |
| GAPDH R | GAAGATGGTGATGGGATTTC | — |
| L-plastin F* | CACTGATCCTCTGGCCATTT | — |
| L-plastin R* | GAAAGAAGGGGTTCCTGGTC | — |
| AP4 F | AGGAGAAAGGGAGAGAG | — |
| AP4 R | TCTTTGAGTAGTCCCTG | — |
| pGL3-∆ARE1 F | CCTTCTCCAAGGTAAAGGAA | PCR-based site-directed deletion |
| pGL3-∆ARE1 R | GTATCTAGTGGTGCCTGGTC | — |
| pGL3-∆ARE2 F | ACAGTTACAACCCCTTGTAT | — |
| pGL3-∆ARE2 R | GGTTCFCACATCCTGGTGGCT | — |
| pGL3-∆ARE3 F | AACATTTAAGGTCAGAAACA | — |
| pGL3-∆ARE3 R | GGTTTATCTACTTCATTAG | — |
| pGL3-∆ERE R | GTTCTGTCAGGTGATTTTG | — |
| pGL3-∆ERE1 R | TCAGACACCTCCTACCACTT | — |
| ChIP-AP4 F | GGCTGATCTTGCCTTGCTAT | ChIP-qPCR |
| ChIP-AP4 R | GAGACAAGGTCCCGCTCTT | — |
| AP4 standard probe | TCAAAGA**CAGCTG**GAGGAGA | EMSA |
| Oligo 1 probe | CAGGGACTACTCAAAGACAGCTGGAGGAGA | — |
| Oligo 2 probe | CTGGAGGAGAAAGGGAGAGAGAAAAATGCT | — |
| Oligo 3 probe | GAAAAATGCTTATAAAGAGGTGGGCAAAAG | — |
| Oligo 4 probe | TGGGCAAAAGAGCGGGACCTTGTCTCAAAA | — |
| Oligo 5 probe | TGTCTCAAAAAAAAAAAAAAAAAAGAGGAA | — |
| Oligo 6 probe | AAAAGAGGAAGTGGTAGGAGGTGTCTGAAT | — |
| Oligo 7 probe | GTGTCTGAATTTCACTGTGACCTGTTCTGT | — |
| Oligo 8 probe | CCTGTTCTGTCAGGTGATTTTTGGTGGGGC | — |
| Oligo 9 probe | TTGGTGGGGCGGGGACATGAAAAAAAAGTT | — |
| Oligo 10 probe | AAAGTTAAAATGTCCTTATAAAGACAAAATCT | — |
| si-AP4#1 F | GAAGGUGCCCUCUUUGCAATT | siRNA |
| si-AP4#1 R | UUGCAAAGAGGGCACCUUCTT | — |
| si-AP4#2 F | GACGCAUGCAGAGCAUCAATT | — |
| si-AP4#2 R | UUGAUGCUCUGCAUGCGUCTT | — |
| si-L-plastin #1 F | CACGAAUGAUCUCUUUAAUTT | — |
| si-L-plastin #1R | AUUAAAGAGAUCAUUCGUGTT | — |
| si-L-plastin #2 F | GACCCGAAGAUUAGUACAATT | — |
| si-L-plastin #2 R | UUGUACUAAUCUUCGGGUCTT | — |
| si-L-plastin #3 F | GUGGACAAGAUCUCAAUGATT | — |
| si-L-plastin #3 R | UCAUUGAGAUCUUGUCCACTT | — |
| sh-AP4 F | GACGCAUGCAGAGCAUCAATT | shRNA |
| sh-AP4 R | UUGAUGCUCUGCAUGCGUCTT | — |

F: Forward primer; R: Reverse primer

**Table S2. Univariate and multivariate Cox regression analyses of overall survival and biochemical disease free survival in PCa patients**

| **Variables** | **Univariate analysis** | | |  | **Multivariate analysis** | | |
| --- | --- | --- | --- | --- | --- | --- | --- |
|  | **HR** | **95% CI** | ***p*-Value** |  | **HR** | **95% CI** | ***p*-Value** |
| **OS** | | | | | | | |
| Age (≤65 vs.>65) | 0.324 | 0.157-0.669 | 0.002** |  | 0.734 | 0.226-2.355 | 0.581 |
| PSA (≤10 vs. >10) | 0.528 | 0.255-1.092 | 0.085 |  | 0.276 | 0.149-0.657 | 0.007** |
| Gleason sore (≤7 vs. >7) | 5.284 | 2.574-10.846 | 0.001** |  | 2.327 | 0.698-6.542 | 0.173 |
| Pathologic stage（≤T2 vs. >T2） | 2.708 | 1.104-6.643 | 0.030* |  | 2.458 | 0.963-6.480 | 0.062 |
| AP4 expression | 2.023 | 0.909-4.507 | 0.084 |  | 1.843 | 0.714-4.759 | 0.206 |
|  | | | | | | | |
| **bDFS** | | | | | | | |
| Age (≤65 vs. >65) | 0.518 | 0.290-0.924 | 0.026* |  | 0.899 | 0.432-1.994 | 0.806 |
| PSA (≤10 vs. >10) | 0.885 | 0.483-1.620 | 0.692 |  | 0.908 | 0.467-1.682 | 0.799 |
| Gleason sore (≤7 vs. >7) | 3.799 | 2.129-6.780 | 0.001** |  | 2.554 | 1.304-5.672 | 0.011* |
| Pathologic stage（≤T2 vs. >T2） | 2.111 | 1.041-4.279 | 0.038* |  | 1.833 | 0.901-3.778 | 0.087 |
| AP4 expression | 2.304 | 1.149-4.622 | **0.019*** |  | 2.034 | 1.012-4.053 | **0.046*** |

Abbreviations: HR = hazard ratio, 95% CI = 95% confidence interval, T stage = tumor stage. Cox regression analysis, **p* < 0.05, ***p* < 0.01, OS: overall survival, bDFS: biochemical disease free survival

**Table S3**. Correlation between AP4 and L-plastin expression and clinicopathologic characteristics of CRPC patients

| **Characteristics** | **Patient frequency (%)** | **ASPC** | | | **CRPC** | |  | **ASPC** | |  | **CRPC** | |  |
| --- | --- | --- | --- | --- | --- | --- | --- | --- | --- | --- | --- | --- | --- |
|  |  | **Low AP4** | **High AP4** |  | **Low AP4** | **High AP4** |  | **Low L-plastin** | **High L-plastin** |  | **Low L-plastin** | **High L-plastin** |  |
| Total cases | 8 |  |  |  |  |  |  |  |  |  |  |  |  |
| Age (yr.) |  |  |  |  |  |  |  |  |  |  |  |  |  |
| <65 | 1 | 0 | 1 |  | 0 | 1 |  | 0 | 1 |  | 0 | 1 |  |
| ≥65 | 7 | 5 | 2 |  | 1 | 6 |  | 4 | 3 |  | 2 | 5 |  |
| Gleason score |  |  |  |  |  |  |  |  |  |  |  |  |  |
| ≤7 | 3 | 1 | 2 |  | 0 | 3 |  | 2 | 1 |  | 1 | 2 |  |
| ＞7 | 5 | 4 | 1 |  | 2 | 3 |  | 3 | 2 |  | 2 | 3 |  |
| Pathologic stage |  |  |  |  |  |  |  |  |  |  |  |  |  |
| ≤T2 | 2 | 1 | 1 |  | 0 | 2 |  | 1 | 1 |  | 0 | 2 |  |
| ＞T2 | 6 | 2 | 3 |  | 1 | 5 |  | 3 | 3 |  | 3 | 3 |  |
| Pathological lymph-node status |  |  |  |  |  |  |  |  |  |  |  |  |  |
| Negative | 3 | 2 | 1 |  | 2 | 1 |  | 1 | 2 |  | 0 | 3 |  |
| Positive | 5 | 4 | 1 |  | 1 | 4 |  | 3 | 2 |  | 1 | 4 |  |

# Supplementary Methods

## *Patients and clinical samples*

A total of 136 paraffin-embedded prostate carcinoma specimens were obtained from patients undergoing radical prostatectomy at Sun Yat-sen Memorial Hospital between February 2005 and July 2015. The definition of CRPC met the criteria of European Association of Urology Guidelines [^1^](#_ENREF_1).The written informed consent was obtained from all patients before sample collection, and approved by Hospital Ethic Review Committees. All samples were snap-frozen in liquid nitrogen and stored at −80 ˚C until required. The diagnosis of prostate carcinoma was confirmed consistently by two certified pathologists with hematoxylin-eosin (H&E) staining. Follow-up data of the prostate carcinoma patients were used for survival analysis. The detailed clinicopathologic characteristics of patients were summarized in Table 1.

## *Cell lines and cell culture*

The prostate carcinoma cell lines (LNCaP and PC-3) used in this study were obtained from American Tissue Type Culture Collection (ATCC, Manassas, VA). Specifically, the hormone-dependent prostate carcinoma cell line LNCaP was maintained in RPMI-1640 medium (HyClone, Logan, UT) with 10% fetal bovine serum (FBS) and hormone-deprived LNCaP cells were maintained in RPMI-1640 medium with 10% charcoal-striped FBS for 7 days. Hormone-independent prostate carcinoma cell line PC-3 was cultured in Dulbecco’s modified Eagle’s medium (DMEM) (HyClone) with 10% FBS. All media were supplemented with 1% streptomycin/penicillin, and the cells were maintained in a humidified atmosphere with 5% CO^2^ at 37˚C.

## *L-plastin promoter constructs*

The promoter sequence of L-plastin was identified and reported (Genbank #：AH002870). Promoter constructs containing the region from -2,197 nt to +118 nt relative to the putative transcription start site of L-plastin gene were PCR-amplified from human genomic DNA using designed primers containing XhoI and Bgl II restriction enzyme sites, respectively. Each fragment was digested with XhoI and Bgl II and purified using the WizardV R SV Gel and PCR Clean-Up System (Promega, Madison, WI) according to the manufacturer’s protocols and then cloned into the pGL3-basic vector (Promega). The fragment with correct L-plastin promoter sequence was cloned into pGL3 and confirmed by sequencing. One of these clones with correct sequence was used for our study.

## *PCR-based site-directed deletion*

The DNA fragment containing the steroid receptor binding sites was created by PCR using specifically designed primers (Table S1) to incorporate the required base pair substitutions using ExSiteTM PCR-Based Site-Directed Mutagenesis kit (Stratagene, La Jolla, CA) according to the manufacturer’s instructions. The template plasmid DNA was alkaline denatured for 15 minutes before PCR procedure. The PCR cycling parameters were as follows: 1 cycle of 95 ˚C for 1 min, 30 cycles of 95˚C for 30 sec, 55˚C for 1 min, and 68˚C for 14 min, a final cycle of 72˚C for 10min. After purification of amplified DNA, linear DNA was ligated at 16 ˚C for 16 hours with T4 DNA ligase. The ligation mixture was extracted twice with phenol: chloroform and precipitated by ethanol. The DNA was digested with 10 units of Dpn I restriction enzyme at 37 ˚C for one and half hour to eliminate the parental plasmid DNA. The Dpn I-digested ligated DNA was then transformed into E. coli XL1-Blue supercompetent cells (Stratagene, La Jolla, CA). This modified procedure successfully produced colonies containing the deletion of ARE1 binding site as confirmed by the sequencing reaction. The same protocol was performed to produce the deletions of ARE1-2, ARE1-3, ARE 1-3 and ERE, and AP4 binding sites. The insert sequences were verified by bidirectional sequencing.

## *Exonuclease directed deletion*

The nested-deletion constructs from pGL3-∆ARE1,2,3-∆ERE in which all hypothetical steroid respond elements were removed were created following the manufacturer's protocols: Mutant 4 was digested with Xho I and Sac I sequentially to generate a 4-nucleotide protruding 3’ terminus. The linear DNA was incubated in exonuclease III reaction buffer at 37 ˚C for 5 min and 300 U of exonuclease III (Stratagene, La Jolla, CA) per pmole of recessed 3’ termini were added into the reaction and immediately incubated in a 30 ˚C water bath. An aliquot of the reaction was taken out every 30 seconds and put into mung bean nuclease (Stratagene, La Jolla, CA) reaction mixture to remove the exposed single-stranded DNA. The resulted set of linear nested DNA fragments have a common terminus corresponding to the 5’ end of the primer with ~200 nucleotides deletion per minute. The aliquots were then analyzed by agarose gel. All the DNA fragments were ligated with T4 DNA ligase and transformed into E. coli competent cells. The corresponding plasmid DNA was extracted and sequenced to confirm the nested deletion.

## *Transfection and Luciferase activity assay:*

LNCaP-AI, LNCaP and PC-3 cells were seeded into six-well plates at a density of 1 x 10^5^ per well in 1 ml of complete medium. The cells were cultured for overnight and temporarily transfected with 1µg each of the mutant constructs of L-plastin promoter produced by site-deleted and nest-deleted mutagenesis using Lipofectamine RNAi Max (Life Technologies, CA, USA). 40 ng of the pRL-TK internal control plasmid was co-transfected into the cells to verify transfection efficiency.

After 24 hours, the culture medium was replaced with the medium mentioned above with or without 10 nmol/ml dihydrotestosterone (DHT) (sigma, St. Louis, MO) for another 24 hours. Cells were lysed and the luciferase activities were measured using the Dual Luciferase Assay System (Promega) and the SpectraMax M5 reader (Molecular Devices, CA). All transfections were carried out in triplicate following manufacturer's instructions.

## *Chromatin immunoprecipitation (ChIP)*

Chromatin immunoprecipitation was conducted with the EZ-Magna ChIP A/G kit (Millipore, Bedford, MA) according to the manufacturer’s instructions. Briefly, 1x 10^6^ cells were used for each reaction. Cells were fixed in 1% formaldehyde at room temperature for 10 min, and the nucleus was isolated with nuclear lysis buffer (Millipore) supplemented with a protease inhibitor cocktail (Millipore). The chromatin was sheared by sonication on ice (total time, 15 min; on time, 30s; off time, 2 min). The sheared chromatin was immunoprecipitated overnight at 4 ˚C with anti-AP4 antibody (N-17) (Santa Cruz Biotechnology, Santa Cruz, CA). Normal mouse IgG was used as a negative control and anti-RNA pol II (Millipore) was used as a positive control antibody. Protein A/G bead antibody/chromatin complexes were washed to remove nonspecific binding. The protein/DNA complexes were reversely cross-linked, and the DNA was purified using spin columns. The purified DNA was detected with qPCR. Prepared samples were amplified using specifically designed ChIP primers (Supporting Information Table S1). PCR conditions for ChIP analysis were as follows: 1 cycle of 94 ˚C for 10 min, followed by 50 cycles of 94 ˚C for 20 sec, 58 ˚C for 30 sec and 60 ˚C for 1 min, 60 ˚C for 5 min, and storage at 4 ˚C. PCR products were separated by electrophoresis in a 1.5% agarose gel and visualized by EB staining.

## *Electrophoretic mobility shift assay (EMSA) and supershift assay*

Nuclear extracts were prepared using a nuclear extraction kit (Active Motif, Carlsbad, CA) according to the manufacturer’s instructions before performing the EMSAs. Ten overlapping oligos and their complementary sequences of the predicted transcription binding site probes within the 206-bp human L-plastin promoter region were synthesized, equimolar of the oligos illustrated in Figure 1. Their complementary oligonucleotides were annealed to prepare double-stranded DNA probes and competitor. For binding assays, 10 μg of LNCaP nuclear extract samples were incubated with purifiedγ-32P-labeled probe using G-50 Sephadex columns (Amersham Biosciences, Piscataway, NJ) at room temperature for 20 min in the binding buffer (Promega). The reaction complexes were resolved on a 5% acrylamide gel and transferred to a positively charged Biodyne B nylon membrane. The membrane was cross-linked under UV light for 15 min. The complexes were visualized using a chemiluminescent nucleic acid detection module (Thermo Scientific). For supershift assays, antibody against AP4 (N-17, Santa Cruz Biotechnology), AP4 (C-18, Santa Cruz Biotechnology), Sp1 (Abcam), HMG and SRY (Santa Cruz Biotechnology) were added to the nuclear extract and incubated on ice overnight prior to the addition of the binding buffer and labeled probe.

## *Microarray analysis*

The PrimeViewTM Human Gene Expression Array (Affymetrix) was used in this study and performed by Gene Tech Corporation (Shanghai, China) according to the manufacturer’s instructions. The arrays were scanned on an Affymetrix GeneChip 7G 3000 Scanner. The microarray data were analyzed using Affymetrix Genechip software, and the raw (.CEL) files generated were analyzed using Expression Console software with Affymetrix default RMA Gene analysis settings. Probe summarization, quality control analysis, and probe annotation were performed per recommended guidelines (Expression Console Software, Affymetrix, Inc. Santa Clara, CA). All primary data in microarray analysis are available at the Gene Expression Omnibus (GEO accession: GSE83140).

## *Cell transfection and viral infection*

LNCaP-AI, LNCaP and PC-3 cells were transfected with small interfering RNA (siRNA) using Lipofectamine RNAi Max (Life Technologies, CA, USA) according to the manufacturer’s instruction. The sequences of the siRNAs were provided in Supporting Information Table S1. All siRNAs were purchased from GenePharma (Shanghai, China). After 48 hr, the efficiency of siRNA knockdown was assessed by qPCR. For stable knockdown of AP4 shRNA (sh-AP4) and normal control RNA (sh-NC) were inserted into the lentiviral vector (pMkO.1-puro vector). After 72 hr transfection, the viral supernatants of 293T cells were collected. Lentiviral particles were concentrated using a LentiX™ Concentrator overnight at 4°C. PC-3 cells (5×10^5^ cells per well) were seeded in six-well culture plates and infected with virus and polybrene for 24 hr. Positive clones were screened with puromycin for two weeks to establish the new stable cell lines: PC-3-sh-AP4 and PC-3-sh-NC.

## *Cell proliferation, colony-formation assay and cell cycle analysis*

The methyl thiazolyl tetrazolium (MTT, Promega) colorimetric assay was used to screen for cell viability. Briefly, 2000 cells were seeded in 96-well plates. Then, 25 ll of 5 mg/ml MTT assay reagent in PBS was added to each well and the cells were incubated at 37 ˚C for 4 hr. The formazan crystals that formed during the incubation were dissolved in DMSO. The absorbance (OD value) at 570 nm was measured using the SpectraMax M5 (Molecular Devices).

For the colony-formation assay, transfected cells were placed in six-well plates and maintained in F12K containing 10% FBS for two weeks. Colonies consisting of 50 or more cells were counted as a clone. The colonies were then fixed with methanol, stained with 0.1% crystal violet (Sigma-Aldrich, Milwaukee, USA) and counted.

For the cell cycle analysis, 48 hr after transfection, cells were harvested and fixed in 70% ice cold ethanol and followed by RNase A treatment, stained with 50 mg/ml of propidium iodide for DNA content analysis in a FACSCaliber BD flow cytometer (BD Biosciences). The data were collected and processed using the BD FACSuite analysis software.

## *Wound healing assay and transwell assay*

Wound-healing assay was used to detect cell migration in vitro. Cells were seeded in 6-well plates and reached 80% confluency in 48 hr. The cell layer was scrapping with a 10-μl sterile pipette tip and washed with three times with PBS to remove the floating and detached cells. Fresh serum-free medium was added, and images were acquired using a microscope at different time points (0, 24 and 48 hr).

Cell migration and invasion assays were performed using transwell chambers (24-well insert, 8 μm, Corning Costar Corp). Cells were harvested at 48 h after transfection and then collected. A total of 1*10^5^ cells were suspended in serum-free medium and placed in the uncoated (migration assay) or 1:4 diluted Matrigel-coated (invasion assay, BD Biosciences, NJ, USA) upper chamber. The lower chamber was filled with 0.6ml of medium containing 20% FBS. After incubation for 24 hr at 5% CO^2^ at 37°C, the cells remaining in the upper chambers were scraped off, and the invading cells were fixed with 4% paraformaldehyde and stained with crystal violet. Five random fields were examined via microscopic observation, and the number of cells was determined.

## *RNA extraction, cDNA synthesis and quantitative real-time PCR analyses*

Total RNA from prostate carcinoma tissues or cultured cell lines was extracted using TRIzol reagent (Life Technologies, Carlsbad, CA), treated with RNase-free DNase (QIAGEN, Germantown, MD) following the manufacturers’ instructions. RNA electrophoresis was conducted to inspect RNA integrity. The cDNA was synthesized using 500ng of total RNA and SuperScript II reverse transcriptase (Invitrogen, San Diego, CA). Quantitative PCR (qPCR) was conducted using the SYBR Green master mix (Roche, CA) and analyzed on a Roche Light-Cycler system (Roche, CA). The relative expression levels were calculated using the 2^-∆∆CT^ method, with GAPDH as an internal control. All specific primers used in the study were listed in Supporting Information Table S1.

## *Protein extraction and Western blot analysis*

Cells were lysed using the protein extraction reagent RIPA buffer (Pierce, Rockford, IL) supplemented with a protease inhibitor cocktail (Roche, Pleasanton, CA, USA). The proteins were extracted and separated by electrophoresis in 10% SDS polyacrylamide gels (SDS–PAGE) and transferred to polyvinylidene fluoride membranes (Millipore) according to the manufacturer’s instructions. Then, the membranes were blocked for 1 hr at room temperature using 5% bovine serum albumin (BSA) and incubated in TBST (Tris buffered saline with 0.05% Tween) containing primary antibodies specific to AP4 (1:1000, Santa Cruz Biotechnology), L-plastin (1:1000, Abcam) and glyceraldehyde-3-phosphate dehydrogenase (GAPDH) (1:1,000, Cell Signaling Technology, Beverly, MA) overnight at 48C. The membranes were incubated with peroxidase-conjugated donkey anti-goat and mouse anti-rabbit immunoglobulin (1:5000, Cell Signaling Technology) as the secondary antibody and were then visualized using an ECL chemiluminescence kit (Pierce).

## *Immunohistochemistry staining and scoring*

Paraffin-embedded samples of primary carcinomas and the xenograft tumor specimens from nude mice were stained for AP4. 5-μm paraffin sections were deparaffinized and hydrated in a graded series of ethanol, followed by microwave treatment in retrieval in citrate buffer (pH = 6.0). Antigen retrieval was performed in 10 mmol/L citrate buffer (pH = 6.0) in a microwave oven for 15 min. The activity of endogenous peroxidases was blocked by the addition of 3% hydrogen peroxide for 10 min at room temperature. Rabbit AP-4 antibody (ab28512, Abcam) was applied overnight at 4°C, and after washing three times in PBS, sections were immunostained with a donkey anti-rabbit secondary antibody (ab1500075, Abcam) for 1 h at 37°C. The slides were incubated with streptavidin-HRP conjugate complex for 45 min at 37°C. The anti-AP4 and anti-Ki67 antibodies (1:1000, Zhongshan Bio-Tech Co. Ltd, Beijing, China) were used to detect the expression of AP4 and Ki67 in nude mice tumors.

For evaluation and grading of AP4 staining results, a scoring criterion was used. Briefly, the staining intensity of AP4 was graded on a scale of 0–3 (0, none; 1, weak; 2, moderate; and 3, strong). The total score was calculated as the product of the scores for the intensity and positive rate of staining. Samples with a staining intensity ≥ 2 were determined as high expression and samples with the staining intensity < 2 were determined as low expression. Cutoff values were determined on the basis of a measure of heterogeneity using the log-rank test with respect to OS and bDFS. Staining was assessed by two pathologists according to the scoring criteria. Cases with discrepancies were jointly reevaluated until a consensus was reached. Images were visualized using a Nikon ECLIPSE Ti (Japan) microscope system and processed with Nikon software.

## *Bioinformatics analysis*

The potential transcription factor binding sites located in the human L-plastin promoter were analyzed using the TRANSFAC ® gene tool software ([http://www.gene-regulation.com](http://www.gene-regulation.com/)), and 0.70 was used as a cut-off value. The datasets from the Oncomine cancer database (<https://www.oncomine.com/resource/main.html>) were selected to determine L-plastin in mRNA expression, which provide fold-change values of gene expression and statistical significance determined by *p* values.

## *Statistical analysis*

All quantitative data are presented as the mean ± standard deviation from at least three independent experiments. The chi-square test (χ^2^ test) for non-parametric variables, and Student’s t test or one-way analysis of variance (ANOVA) for parametric variables was used (two tailed). Analysis was performed using GraphPad InStat Software (GraphPad Software, Inc., San Diego, CA). The differences between groups of AP4 expression in bladder cancer tissues were analyzed using the χ^2^ test. Biochemical failure of prostate cancer was defined as any two consecutive increases in PSA or a single PSA increase >10% in magnitude with a value of 0.4 ng/ml or greater. The date of PSA failure was designated as the midpoint between the date of nadir PSA and the date of the initial increase. Overall survival (OS) and biochemical disease-free survival (bDFS) were evaluated using the Kaplan–Meier method. All statistical analyses were conducted using SPSS v.16.0 (SPSS Inc., Chicago, IL), and *p* values <0.05 were considered statistically significant. An asterisk represents a *p* values <0.05 in all figures.

**Reference**

1. Mottet N, Bellmunt J, Bolla M, Briers E, Cumberbatch MG, De Santis *M, et a*l. EAU-ESTRO-SIOG Guidelines on Prostate Cancer. Part 1: Screening, Diagnosis, and Local Treatment with Curative Intent*. European urolo*gy 2016.
